# Sex Differences in Renal Mitochondrial Respiration and H_2_O_2_ Emission in Young Dahl Salt-Sensitive Rats

**DOI:** 10.1093/function/zqaf045

**Published:** 2025-10-07

**Authors:** Chun Yang, Devanshi D Dave, Sri Rahavi Boovarahan, Satoshi Shimada, Aron Geurts, Ranjan K Dash, Allen W Cowley

**Affiliations:** Department of Physiology, Medical College of Wisconsin, Milwaukee, WI 53226, USA; Department of Biomedical Engineering, Medical College of Wisconsin, Milwaukee, WI 53226, USA; Department of Biomedical Engineering, Medical College of Wisconsin, Milwaukee, WI 53226, USA; Department of Physiology, Medical College of Wisconsin, Milwaukee, WI 53226, USA; Cardiovascular Research Center, Medical College of Wisconsin, Milwaukee, WI 53226, USA; Department of Physiology, Medical College of Wisconsin, Milwaukee, WI 53226, USA; Cardiovascular Research Center, Medical College of Wisconsin, Milwaukee, WI 53226, USA; Department of Physiology, Medical College of Wisconsin, Milwaukee, WI 53226, USA; Department of Biomedical Engineering, Medical College of Wisconsin, Milwaukee, WI 53226, USA; Cardiovascular Research Center, Medical College of Wisconsin, Milwaukee, WI 53226, USA; Department of Physiology, Medical College of Wisconsin, Milwaukee, WI 53226, USA; Cardiovascular Research Center, Medical College of Wisconsin, Milwaukee, WI 53226, USA

**Keywords:** sex differences, mitochondrial bioenergetics, reactive oxygen species, renal metabolism, proximal tubular transport, oxidative stress

## Abstract

Sexual dimorphism profoundly influences physiology, disease susceptibility, and therapeutic responses, yet its effects on kidney mitochondrial function remain unclear. We hypothesized that sex differences in kidney mitochondrial function would parallel those in other organs, with females exhibiting greater oxidative capacity and lower oxidative stress. To test this, we measured oxidative phosphorylation (OXPHOS) kinetics and hydrogen peroxide (H_2_O_2_) emission in cortical and outer medullary (OM) mitochondria isolated from young male and female Dahl salt-sensitive (SS) rats maintained on a low-salt diet. Unexpectedly, male cortical mitochondria showed significantly higher O_2_ consumption during ATP synthesis (OXPHOS) than females when fueled by either complex I- or complex II-linked substrates. Cortical H_2_O_2_ emission was also greater in males, under both forward and reverse electron transport fueled by succinate. This was accompanied by increased Complex IV protein abundance without changes in mitochondrial DNA copy number or dynamics markers. In the OM, both mitochondrial respiration and H_2_O_2_ emission exceeded cortical levels, but showed no sex differences. Analysis of kidney transporter protein abundance revealed a sex-specific “downstream shift” in nephron transport, with males exhibiting greater proximal tubule (PT) sodium reabsorption potential and reduced distal transport capacity. Elevated cortical OXPHOS activity in males likely supports these higher PT energy demands. Thus, sex differences in renal mitochondrial function diverge from other organs, reflecting kidney-specific energetic priorities that override systemic maternal inheritance and sex hormone influences. Enhanced cortical H_2_O_2_ emission in males may underlie their heightened susceptibility to kidney injury and salt sensitivity.

## Graphical Abstract

**Graphical Abstract:**
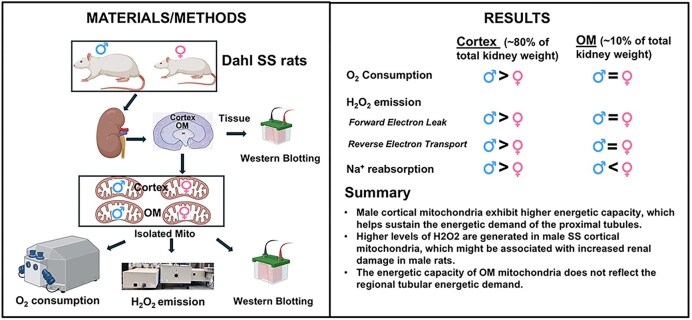

## Introduction

Sex-specific differences extend beyond physical traits, exerting a profound influence on numerous physiological processes. These include, but are not limited to, drug metabolism, immune function, adipose tissue distribution, and overall metabolism. Consequently, observable sex differences emerge in the manifestation of diseases, the organ-specific patterns of injury across various conditions, and the efficiency of therapeutic interventions.^1-3^

Such differences are consistently evident in the onset and progression of metabolic, cardiovascular, and kidney diseases, including diabetes and hypertension.^4-6^ Mitochondrial dysfunction is a common feature of these conditions and has been implicated in differential susceptibility between sexes.^7,8^ A comprehensive understanding of the mechanisms by which mitochondrial function contributes to sex-specific physiology and disease is therefore essential.

The Dahl salt-sensitive (SS) rat serves as a well-established model for studying the renal mechanism underlying SS hypertension.^9,10^ Studies have consistently documented sex-specific hemodynamic responses in SS rats under both basal conditions and during salt-induced hypertension. Although baseline blood pressure does not differ significantly between sexes from young and aged SS rats on a normal salt diet, male rats exhibit more pronounced age-related renal injury, including increased urinary protein excretion and reduced glomerular filtration rate.^11^ In contrast, female rats develop high blood pressure (BP) more slowly and with less severity when exposed to a high salt (HS) diet and exhibit less renal injury.^12-15^ Similarly, in a high-fat diet-induced obesity model, female SS rats showed attenuated renal injury and lower macrophage infiltration compared to males.^16^ These findings in SS rats parallel clinical observations that women are generally protected from cardiovascular disease, chronic kidney disease, and hypertension, which are more prevalent in age-matched men than women before menopause.^17^ However, the mechanisms underlying this female protection remain incompletely understood.

Mitochondrial sexual dimorphism has emerged as a key factor in these differences. In most tissues, female mitochondria exhibit enhanced respiratory function, improved bioenergetic efficiency, and greater antioxidant capacity, along with lower reactive oxygen species (ROS) compared to male mitochondria (see Table 1 in reference^7^). These sex-based differences are thought to arise from the maternal inheritance of mitochondria and the regulatory influence of sex hormones on mitochondria energy metabolism. Sultanova et al.^17^ provide a comprehensive review of sex-specific regulation of renal mitochondrial function, highlighting the roles of sex hormones, calcium signaling and iron handling. Together, these findings suggest that mitochondrial function may contribute to the differential susceptibility to renal and cardiovascular injury observed between male and female SS rats.

**Table 1.** Comparison of Animal Characteristics and Mitochondrial Isolation from the Cortex and OM of Male and Female Rats ​​​​​​

Animal information and mitochondria isolation							
	Male			Female			*P* value
	Mean	SEM	N	Mean	SEM	N	TTEST
Animal information							
Age (weeks)	7.2	0.06	8	7.2	0.06	8	>0.9999
Body weight (g)	225.1	5.13	8	168.1	4.29	8	<0.0001
Kidney weight (g)	1.99	0.05	8	1.59	0.04	8	<0.0001
Kidney/100 g body weight	0.884	0.01	8	0.925	0.01	8	0.03
Cortical mitochondria							
Input weight (g)	1.48	0.04	8	1.13	0.03	8	<0.0001
Total mitochondria yield (mg)	6.9	0.37	8	5.1	0.19	8	0.00
Isolation efficiency (‰)	4.7	0.31	8	4.6	0.20	8	0.71
OM mitochondria							
Input weight (g)	0.19	0.01	8	0.19	0.01	7	0.74
Total mitochondria yield (mg)	0.34	0.03	8	0.37	0.05	7	0.65
Isolation efficiency (‰)	1.8^a^	0.18	8	1.9^a^	0.15	7	0.60

a: *P* < 0.001 versus cortical mitochondria. Higher cortical tissue input from male rats resulted in a higher mitochondria yield, with no sex difference in isolation efficiency.

Sex-specific differences in kidney nephron structure and function are well established in both rodents and humans. These differences are reflected in multiple domains, including gene expression,^18-20^ protein abundance,^21^ metabolomic profiles,^3,4^ transporter activity,^15,22-26^ and overall kidney function and disease susceptibility.^6,27-29^ Computational models of Layton and colleagues estimate that females reabsorb only half of the filtered load in the proximal tubules (PTs), compared to two-thirds in males,^30^ a prediction supported by endogenous lithium clearance studies, which show greater volume flow from the proximal to distal nephron segments in females.^31^ This lower proximal Na^+^ reabsorption is attributed to reduced activity of the Na^+^/H^+^ exchanger (NHE3) in females’ PT and is compensated by enhanced sodium and water reabsorption in distal segments, a “downstream shift” that may serve as an adaptive mechanism during pregnancy and lactation.^22^

This segmental redistribution has important bioenergetic and redox implications. Because Na^+^ transport depends on energy-intensive basolateral Na^+^/K^+^-ATPase activity, the greater proximal Na^+^ absorptive load in males imposes a higher ATP demand on the cortical nephron. In the Dahl SS rat, this burden becomes especially pronounced during high-salt intake, which increases the filtered Na^+^ load and requires ∼99% resorption by the tubules, primarily in the PT (∼65%) and medullary thick ascending limb (mTAL, ∼25%).^32-36^ Meeting this metabolic challenge necessitates substantial mitochondrial ATP production, potentially exceeding what can be attributed to tissue mass alone. This prompted us to investigate whether sex-specific differences in mitochondrial function align with these differential energetic demands.

In the present study, since mitochondria are maternally inherited, we hypothesized that sex differences in mitochondrial function in the kidney would parallel those observed in other organs, where females often display higher mitochondrial respiratory capacity and a premenopausal protective advantage. To test this, we assessed mitochondrial bioenergetics and redox activity in the renal cortex and outer medulla (OM) of male and female SS rats by measuring substrate-dependent oxygen consumption rates (OCR or JO_2_) and the associated hydrogen peroxide (H_2_O_2_) emission. This approach enabled a comprehensive evaluation of both mitochondrial energy production and oxidative stress, two critical factors implicated in salt-induced hypertension and renal injury in this model.^37-39^ Our findings did not support the prediction that renal mitochondrial sex differences mirror those in other organs, suggesting that kidney-specific physiological demands, rather than systemic maternal inheritance effects, predominate in shaping mitochondrial bioenergetics. Higher cortical H₂O₂ emission in males may contribute to greater susceptibility to kidney injury and salt sensitivity.

## Materials and Methods

### Animals

Male and female Dahl SS rats (SS/JrHsd Mcwi; SS) were obtained from the Medical College of Wisconsin (MCW) and maintained on a 0.4% NaCl rodent chow (AIN-76 purified; Dyets No. 113755) after weaning. All animal procedures and experimental protocols were approved by the MCW Institutional Animal Care and Use Committee (IACUC, no. 851). Rats were anesthetized with Inactin (100 mg/kg i.p.), and kidneys were harvested. The cortex and OM regions were dissected and maintained at 4°C before the mitochondrial isolation procedure.

### Mitochondrial Isolation and Quantification

Kidney cortex and OM mitochondria were isolated using a modified differential centrifugation method^40,41^ at 4°C. Approximately 1.5 g/1.2 g (male/female) of whole cortex tissues and 0.2 g of OM tissue from both kidneys were minced in ice-cold Isolation Buffer (IB: 50 mM sucrose, 5 mM KH_2_PO_4_, 5 mM MOPS, 1 mM EGTA, 0.1% BSA, pH 7.15). Cortex tissue was homogenized in 10 mL IB for 15 s, and OM tissue in 2 mL IB for 5 s. The volume was adjusted to 15 mL (cortex) or 2 mL (OM) with IB, and homogenates were centrifuged at 600 *g* for 10 min. The supernatant was collected and centrifuged at 12 000 *g* for 15 min. Pellets were resuspended in 15 mL (cortex) or 2 mL (OM) IB and centrifuged again at 12 000 *g* for 15 min. The final mitochondrial pellets were resuspended in 400 μL (cortex) or 50 μL (OM) IB and kept on ice until used for studies. Protein concentrations were determined using the Bio-Rad Quick Start Bradford Assay Kit with BSA as a standard, measured with a Thermo Scientific NanoDrop OneC spectrophotometer. Typical protein concentrations were approximately 9-12 µg/µL (cortex) and 4-7 µg/µL (OM).

### Mitochondrial Oxygen Consumption and ADP Phosphorylation (OXPHOS) Analysis

Mitochondrial O_2_ consumption was assessed using a high-resolution Oxygraph-2k (O2k) respirometer (Oroboros Instruments) equipped with DatLab 7 software, following established protocols.^40-42^ Chambers (2 mL volume) were filled with Respiration Experimental Buffer (EB; 130 mM KCl, 5 mM K₂HPO_4_, 20 mM MOPS, 1 mM EGTA, 0.1% BSA, pH 7.15) and calibrated to 210 µm O₂ at 37°C with stirring at 750 rpm.

Mitochondria isolated from male and female renal tissues were loaded side-by-side into each of the 2 mL Oroboros respiratory chambers (0.4 mg for cortex mitochondria; 0.1 mg for OM mitochondria). A near-zero O_2_ consumption baseline (State 1) was established prior to substrate addition. Respiration was initiated by the addition of substrate combinations at saturating concentrations: pyruvate + malate (5 mM/2.5 mM, PM), succinate (10 mM), or glutamate + malate (5 mM/2.5 mM, GM), inducing basal (State 2) respiration. ADP was then added (200 µm for cortex mitochondria; 100 µm for OM mitochondria) to stimulate State 3 respiration and kept in monitoring after it reached to State 4 respiration. Changes in O_2_ concentration (µm) within the chambers and O_2_ consumption rate (OCR; JO₂) were continuously recorded. Data were collected as pmol O₂/sec/mL chamber volume and reported as nmol O₂/min/mg mitochondrial protein.

Isolated mitochondria were stored on ice during the experiments, and all tests finished within 4 h after harvest, before the drop of respiration.

### H_2_O_2_ Emission Assay

**Figure 1.**
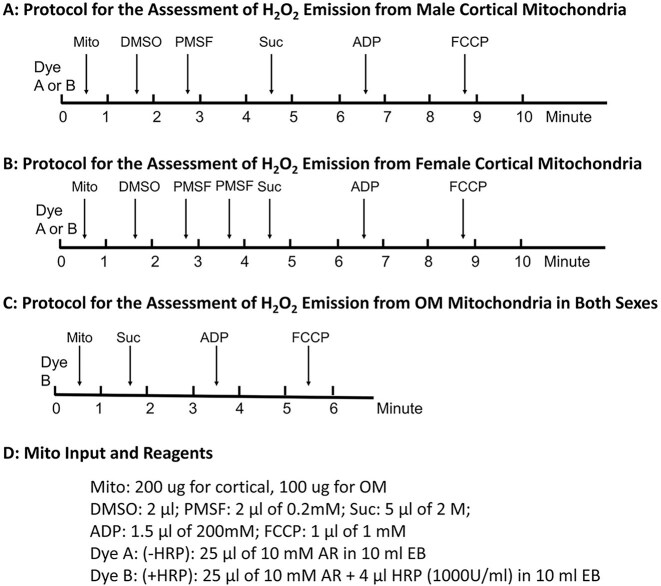
Protocols for assessing H_2_O_2_ emission from cortical and outer medulla (OM) mitochondria. (A) Protocol used to assess H_2_O_2_ emission from cortical mitochondria in male SS rats. (B) Protocol used to assess H_2_O_2_ emission from cortical mitochondria in female SS rats. (C) Protocol for assessing H_2_O_2_ emission from OM mitochondria in both male and female rats. (D) Mitochondrial input and a list of all reagents used in the protocols depicted in panels (A-C).

Mitochondrial H₂O₂ emission was measured using a Horiba PTI QuantaMaster 8000 spectrofluorometer. Assays were conducted in cuvettes at 37°C in EB Buffer containing 25 µm Amplex Red (10-acetyl-3,7-dihydroxyphenoxazine; AR) and 0.4 U/mL horseradish peroxidase (HRP). Excitation and emission wavelengths were set at 575 nm and 583 nm, respectively. The assay relies on HRP-catalyzed oxidation of AR by H₂O₂ to produce fluorescent resorufin. H₂O₂ generation was stimulated following respiration protocol by sequential addition of succinate (10 mM), ADP (300 µm), and FCCP (1 µm) (Figure 1). H₂O₂ emission rates were calculated from the slope of resorufin fluorescence following each addition. Fluorescence intensity was calibrated daily against H₂O₂ standards generated by sequential addition of known concentrations of H₂O₂ to EB (AR + HRP) without mitochondria. Standard curves exhibited strong linearity (R² > 0.97) across the fluorescence range in the tests. A different protocol was employed to investigate the reverse electron transport (RET)-induced H₂O₂ production in succinate-driven H₂O₂ emission at State 2 respiration. Mitochondria were pretreated with PMSF (phenylmethylsulfonyl fluoride, a serine protease inhibitor) before loading to the cuvette and rotenone (10 µm) was applied prior to succinate (10 mM).

A strong HRP-independent resorufin fluorescence was observed in cortical mitochondrial samples from both sexes as has been observed by others in Sprague Dawley rat liver mitochondria.^43^ The artifact was attributed to carboxylesterase-mediated conversion of AR to resorufin independent of H₂O₂ or HRP. To correct this, a control run without HRP (-HRP) was performed prior to each experimental run with HRP (+HRP) to optimize the inhibition of the background resorufin signal. The optimal concentration of the serine protease inhibitor PMSF was determined by identifying the lowest dose required to completely suppress the fluorescence changes after the addition of succinate in the -HRP control run. This background fluorescence was proportional to mitochondrial input, and was significantly higher in females compared to males, so higher PMSF concentrations were required for female cortical mitochondria (800 µm) compared to males (400 µM). For each sample, H₂O₂ emission was calculated as the difference between the fluorescence slopes of the +HRP and -HRP runs at each respiration status. Note that State 4 respiration was not reached with the ADP concentrations used for a stable measurement of plateau State 3 respiration- associated H₂O₂ emission. Measurements of H₂O₂ emission were conducted for isolated mitochondria from the kidney cortex and OM following the experimental timelines (Figure 1). To ensure rigor and reproducibility, our refined protocol incorporated: (1) HRP-negative (-HRP) controls to assess the best inhibition of background fluorescence; (2) technical replicates of HRP-positive (+HRP) assays; (3) paired processing of male and female samples; and (4) the use of daily H₂O₂ standard curves for calibration. Validation experiments confirmed that these optimized conditions effectively eliminated background signals across sexes, enabling accurate measurements of H₂O₂ emission in isolated cortical mitochondria under diverse conditions (substrates, ADP, and FCCP).

### Western Blotting

Western blots were performed on isolated mitochondria and tissue homogenates as described previously.^44^ The following antibodies were used: Total OXPHOS Rodent WB Antibody Cocktail ab110413 (Abcam), pNCC T53 (Phosphosolutions), anti-ENaC α (StressMarq Biosciences), anti-ENaC γ (StressMarq Biosciences), AQP1 (Abcam), and GAPDH (Santa Cruz), Antibodies to mitochondrial fission and fusion dynamics marker proteins DRP1, OPA1, Tom20, and Mitofusion2 are all from Cell Signaling Technology. NKCC2, pNKCC2 T96, and pNKCC2 T101 were obtained from the University of Dundee.

### Na-K-ATPase Activity Assay

Total ATPase/GTPase activity was measured using home-prepared reagents, utilizing colorimetric assessment of free phosphate, adapted from Moraes.^45^ Frozen kidney cortical tissues were homogenized in histidine buffer (250 mM Mannitol, 30 mM L-histidine, 5 mM EGTA, 0.1% Deoxycholic acid). The supernatant, after 400 *g* for 5 min centrifugation, was further spun down at 15 600 *g* for 40 min at 4°C. The pellets (membrane fractions) were suspended in histidine buffer and protein quantification was carried out using Protein Assay Solution (Bio-Rad Cat#5000 113). Samples containing 2.5 µg of kidney cortical membrane fraction protein were mixed with assay buffer (120 mM NaCl, 4 mM MgCl_2_, 100 mM Tris-HCl) and 10 µL of 4 mM ATP (Sigma-Aldrich) to a total of 50 µL. Samples were incubated at 37°C for 10 min. Color reagent (BIOMOL Green reagent BML-AK111) was added, and samples were incubated for 30 min at RT. Absorbance at 620 nm was measured using a spectrometer (Spectra Flour Plus; TECAN). Ouabain-sensitive ATPase activity was measured similarly, except samples containing 2.5 µg of kidney cortical membrane fraction protein were pre-incubated with 10 µL of 3 mM ouabain (Sigma-Aldrich) for 30 min at 37°C prior to incubation with ATP. Samples were run in triplicate and averaged. The difference between total ATPase activity and ATPase activity in the presence of ouabain was taken as Na-K-ATPase activity.

### Mitochondrial DNA Copy Number Assessment

Total DNA was purified from frozen cortical tissues. DNA quantity and purity was determined by spectrometric analysis. The total DNA showed a higher purity (A260/280 > 1.8) and was stored at 4°C according to standard procedures. The primers used in this assessment include primers for nuclei genome PDE4b site (cAMP-specific 3“,5”-cyclic phosphodiesterase 4B) nPDE4: f GTTCCCGCCTTCTTC CTCTG, r GTTTGCTTGCCGACTCCTTG (chr5:122560457-122560601), Subunit 2 of cytochrome c oxidase gene site on mitochondria genome mitoCox2: f TGGCTTACAAGACGCCACAT, r TGGGCGTCTATTGTGCTTGT (mito:7028-7183); 12S ribosomal RNA gene site on mitochondria genome mito12Sr f: GTGAAATCAACAACCCGCCC, r ATAGTCACCCCCAGGACGAA (mito:278-347). Amplification was performed on QuantStudio 6 Flex (Applied Biosystems) anneal at 60°C. Data were analyzed as mitochondrial DNA copy number per 1000 copies of the nuclear genome through the delta-delta-Ct method and presented after normalization to the average of males.

### Data Processing and Statistical Analysis

MS Excel and GraphPad Prism 10 (version 10.2.3) were used for data processing, graph generation, and statistical analysis. Unpaired Student’s *t*-tests were used for isolated mitochondria data when males and females were compared. Two-Way ANOVA Fisher’s LSD test was applied to the comparisons of Cortex and OM. *P* < 0.05 was considered significant.

## Results

### Male-Female Trait Differences and Kidney Mitochondrial Yields

Seven-week-old Dahl SS rats exhibited higher body weight and kidney weight in males compared to females. In contrast, the kidney weight-to-body weight ratio was higher in females. To maintain the integrity of the tissue, we used the remaining cortical tissue after removing the medullary region for the isolation of cortical tissue mitochondria. Consistent with larger kidneys, the total amount of cortex tissue input for mitochondrial isolation was greater in males, resulting in a higher total mitochondrial yield from the cortical tissue in males. No significant sex differences in tissue input or mitochondrial yield were observed in the OM tissues. The mitochondrial isolation efficiency, as indicated by the ratio of mitochondrial yield to tissue input, was the same between sexes for both cortex and OM samples, supporting the comparability of male and female mitochondrial samples. Notably, this ratio was higher in cortex tissue than in OM tissue in both sexes, suggesting a potentially higher mitochondrion density in the cortex tissue. These data were shown in Table 1.

### Mitochondrial Oxygen Consumption in the Cortex and OM

**Figure 2.**
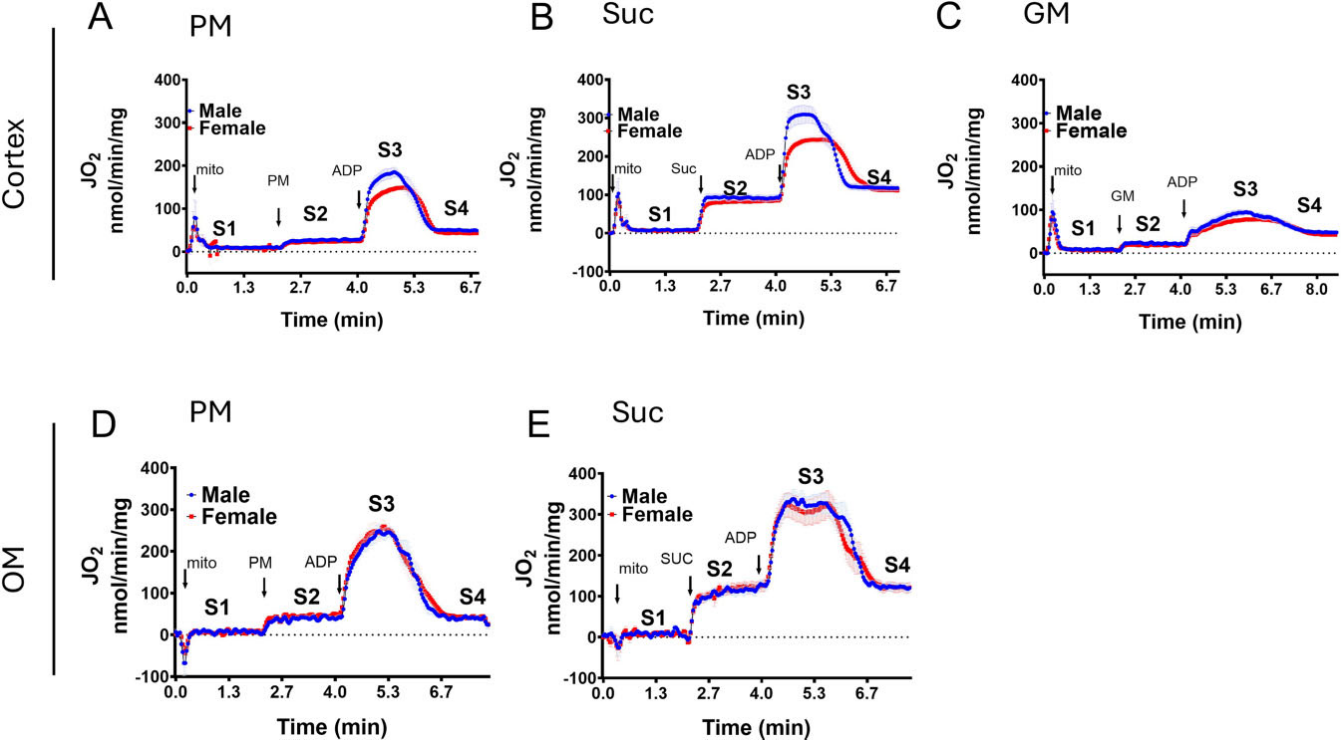
Time courses and sex differences in substrate-dependent respiration of renal cortical and OM mitochondria of SS rats. Shown are the O_2_ consumption rates (JO_2_) of isolated mitochondria from the renal cortex (A-C) and OM (D-E) of male and female SS rats during different states of respiration. Mean JO_2_ values are shown as lines, with shaded areas representing SEM (*n* = 5). Notable sex differences were evident in the cortical mitochondrial respiration across different respiratory substrates.

Figure 2 shows the sex-specific and substrate-dependent time courses of O_2_ consumption rates (JO_2_) during different states of respiration for cortical (Figure 2A-C) and OM mitochondria (Figure 2D, E). In both tissues, succinate (Suc), the FADH₂ (Complex II)-linked substrate, elicited the highest JO_2_ in all states of respiration (Figure 2B and E), in comparison to the NADH (Complex I)-linked substrates pyruvate + malate (PM) and glutamate + malate (GM) (Figure 2A, C, and D). The addition of a substrate induced H^+^-leak state (State 2; S2) respiration, which was highest with succinate. The addition of ADP (200 µm for 0.2 mg/mL cortex mitochondria; 100 µm for 0.05 mg/mL OM mitochondria) induced State 3 respiration (active or phosphorylating respiration; S3), characterized by an initial increase and subsequent decrease in JO_2_ due to saturated but limited ADP availability. PM and Suc induced a sharp increase and decrease in respiration traces in both the tissues in response to ADP (Figure 2A, B, D, and E), while GM resulted in a shallow increase and decrease in respiration in the cortex (Figure 2C; not studied in the OM due to limited mitochondrial sample). Despite both PM and GM being complex I-linked (NADH-linked) substrates and the same addition of ADP, the differences in the pattern of time course respiration during State 3 respiration with these substrates indicated different activities of the pyruvate and glutamate metabolism in the cortex. Tissue-specific respiration was also observed from the shape of ADP-dependent respiration curves induced by succinate (Figure 2B and E). JO_2_ traces in the OM samples were more fluctuating at the plateau phase than those from the cortex tissue. These tissue-specific respiration patterns may reflect differences in the substrate metabolism pathways in these tissues. Oxygen consumption eventually stabilized after ADP depletion, reaching State 4 respiration (S4), a post-phosphorylation (resting) state. State 4 respiration was higher than State 2 in both sexes and with all substrates. These tissue-specific and substrate-dependent dynamic respirations were consistent with previous findings.^40,41^ A clear separation of State 3 respiration curves between males and females was also observed in cortical mitochondria, while no such apparent separation was evident in State 2 or State 4. In addition, no clear separation in JO_2_ profiles was evident in the OM mitochondria for males and females in any state of respiration.

**Figure 3.**
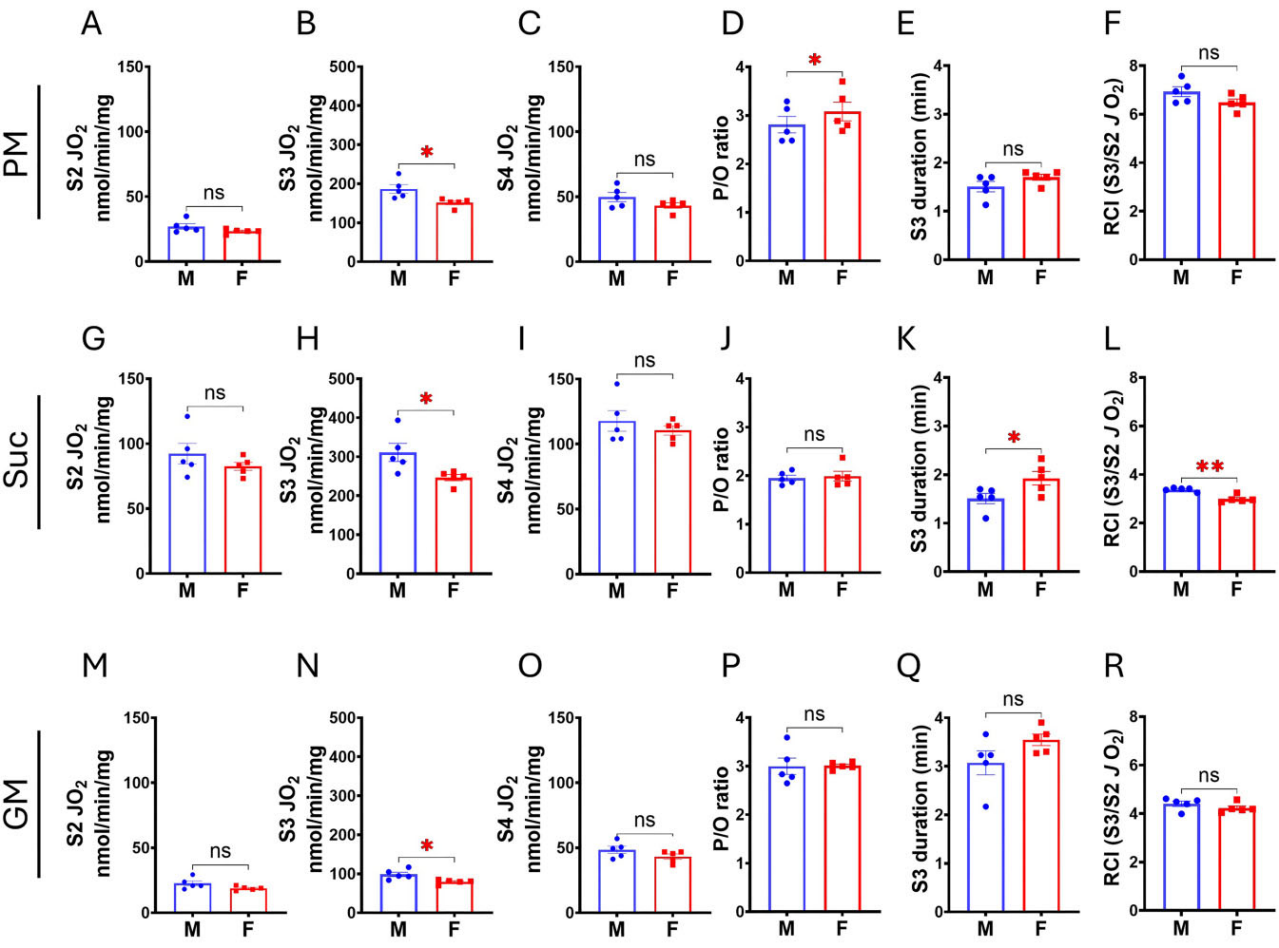
Male SS rats exhibit higher ADP phosphorylation-linked mitochondrial respiration (JO_2_) in the renal cortex. (A, G, M) State 2 (S2) JO_2_ with PM (pyruvate + malate), Suc (succinate), or GM (glutamate + malate) as substrates. (B, H, N) State 3 (S3) JO_2_ following ADP stimulation (200 µm). (C, I, O) State 4 (S4) JO_2_ post-ADP phosphorylation. (D, J, P) P/O ratio indicating oxidative phosphorylation (OxPhos) coupling efficiency. (E, K, Q) S3 duration representing ADP addition to S4 transition time. (F, L, R) Respiratory control index (RCI; S3/S2 JO_2_ ratio) indicating mitochondrial integrity. Data are shown as mean ± SEM and individual values (*n* = 5 per group). Student’s *t*-test used. * *P* < 0.05, **P < 0.01.

**Figure 4.**
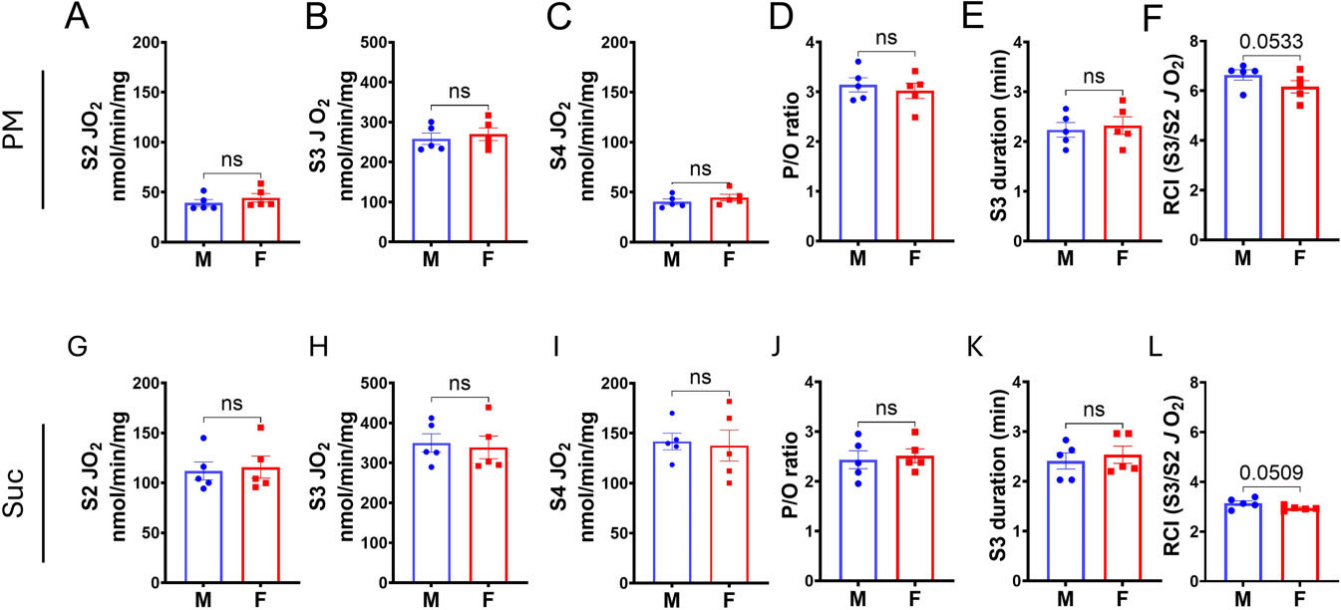
Absence of sex-specific effects on renal OM mitochondrial respiration (JO_2_) in SS rats. (A, G) State 2 (S2) JO_2_ with pyruvate + malate (PM) and succinate (Suc). (B, H) State 3 (S3) JO_2_ following 100 µm ADP stimulation. (C, I) State 4 (S4) JO_2_ post-ADP phosphorylation. (D, J) P/O ratio indicating oxidative phosphorylation (OxPhos) coupling efficiency. (E, K) S3 duration (ADP to S4 transition time). (F, L) Respiratory control index (RCI; S3/S2 JO_2_ ratio), reflecting mitochondrial integrity. Data are shown as mean ± SEM and individual values (*n* = 5 per group). Statistical comparisons were made using Student’s *t*-test

O_2_ consumption parameters for males and females, derived from the curves in Figure 2, are presented in Figure 3 (cortex mitochondria) and Figure 4 (OM mitochondria). State 2 and State 4 JO_2_ were calculated as average rates during the respective periods, while State 3 JO_2_ was defined as the maximum rate following ADP addition. State 3 duration is the time from ADP addition to the start of stable State 4 respiration and was used for ADP/O ratio (P/O ratio) calculation. The P/O ratio (moles ATP produced per moles of oxygen atom consumed) was calculated by dividing the total ADP added by 2 times the total O_2_ consumed (corrected for State 2 JO_2_) during State 3. The Respiratory Control Index (RCI) was calculated as the ratio of State 3 JO_2_ to State 2 JO_2_. These are important respiratory parameters to understand mitochondrial function with different substrates, characterizing mitochondrial H^+^ leak state (States 2 and State 4 JO_2_), phosphorylation state (State 3 JO_2_ and duration), OXPHOS efficiency (P/O ratio), and mitochondrial functional integrity (RCI) of cortex and OM tissues in males and females.

Figure 3A-F show parameters for cortical mitochondria respiration with pyruvate + malate (PM) as the substrate, G-L with succinate (Suc), and M-R with glutamate + malate (GM). Consistent with the O_2_ consumption curves in Figure 2, quantitative analysis of the mitochondrial respiratory parameters showed sex dimorphism in the renal cortex. State 3 JO_2_ were significantly higher in male cortical mitochondria across all substrates (Figure 3B, H, and N). State 2 and State 4 JO_2_ tended to be higher in males with PM and GM, although these differences were not statistically significant (Figure 3A, C, M, and O). State 3 duration was significantly longer in females oxidizing succinate (Figure 3K), with a longer but not statistically significant trend observed with PM and GM (Figure 3E and Q). Despite equal mitochondrial isolation efficiency and identical assay conditions, RCI was significantly higher in males with succinate (Figure 2L), with a higher, but not statistically significant, trend observed with PM and GM (Figure 2F and R) in males.

Figure 4A-F and G-L show the respiratory parameters for OM mitochondria induced by PM and succinate, respectively. In OM mitochondria, oxidizing with complex I-linked PM and complex II-linked succinate, no significant sex differences were observed in any calculated parameters (S2: Figure 4A and G; S3: Figure 4B and H; S4: Figure 4C and I; P/O ratio: Figure 4D and J; S3 duration: Figure 4E and K). A potential, but not statistically significant, trend toward higher RCI in males was observed with both PM and succinate substrates, with p-values of 0.0533 and 0.0509, respectively (Figure 4F and L).

### H₂O₂ Emission in the Cortical and OM Mitochondria

**Figure 5.**
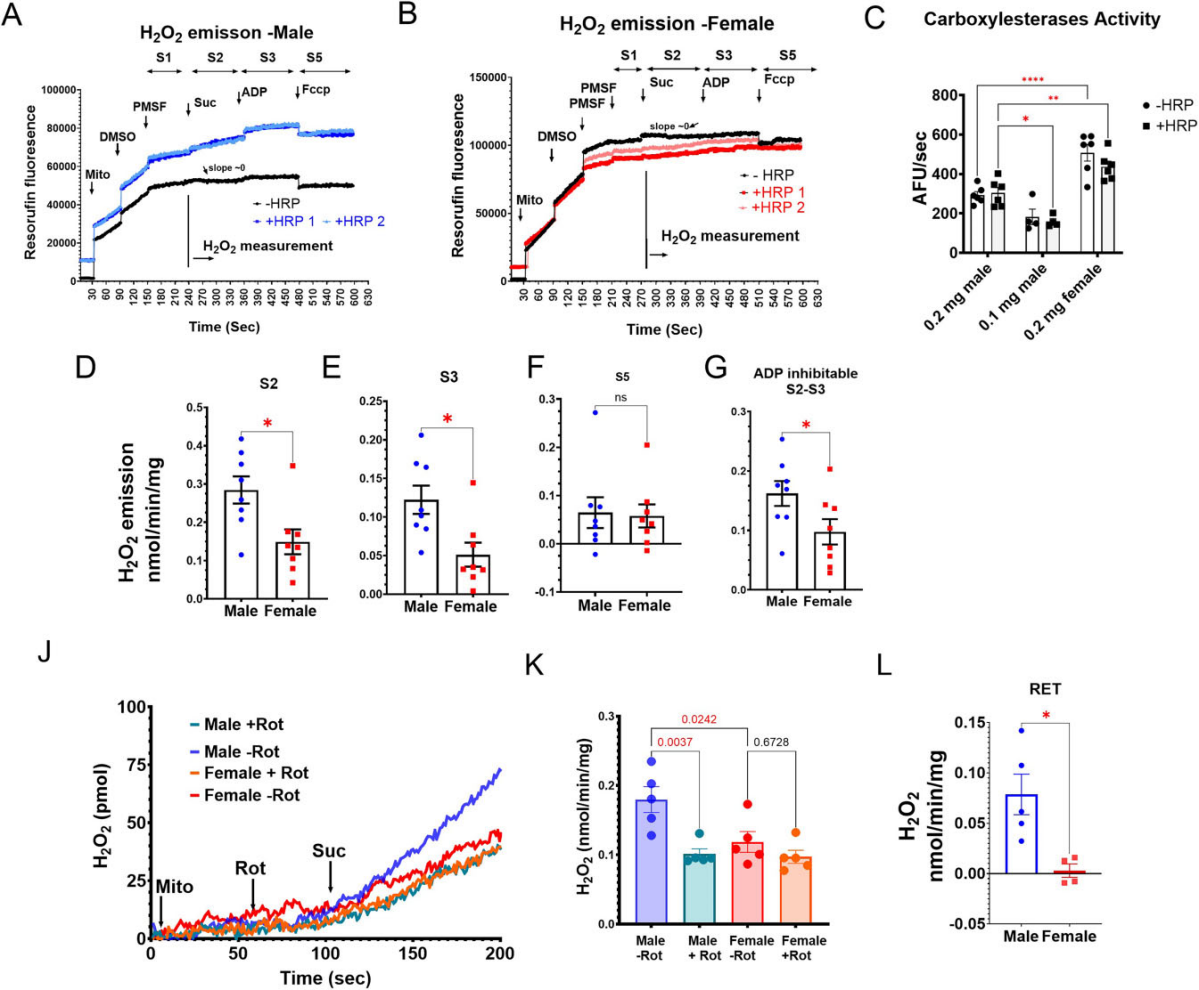
Sex-specific renal cortical mitochondrial H₂O₂ emission, showing elevated mitochondrial H₂O₂ emission from renal cortex in male SS rats originates from two primary sources. (A, B) Representative traces of resorufin fluorescence from renal cortical mitochondria with different perturbations in the presence of Amplex Red (AR) with (+) and without (-) horseradish peroxidase (HRP) in male (A) and female (B) SS rats. (C) Carboxylesterase activity estimated from baseline resorufin fluorescence prior to PMSF addition (S1). (D-G) Mitochondrial H_2_O_2_ emission rates at succinate-supported (S2, D), ADP-phosphorylation (S3, E), and FCCP-uncoupling (S5, F), along with ADP-inhibitable H_2_O_2_ emission (S2-S3, G). (H): Representative traces of H_2_O_2_ emission changes when Rotenone was added before the addition of Suc. Mitochondria were mixed with PMSF before the administration, and H_2_O_2_ concentration was set as 0. (I): Quantification of S2 H_2_O_2_ emission with and without Rotenone in both male and female cortical mitochondria. (J): Reverse electron transport (RET) driven H_2_O_2_ emission. Data are shown as mean ± SEM and individual values (*n* = 5-7 per group). Statistical significance was determined using a Student’s *t*-test. *P < 0.05; **P < 0.01, ^****^  *P* < 0.0001. Data reveal significantly higher H_2_O_2_ emission in males at S3 and from RET.

As detailed in the “Materials and Methods” section, a significant resorufin fluorescence was observed with the addition of fresh cortical mitochondria to the Respiration Experimental Buffer (EB) containing AR with or without Horseradish Peroxidase (±HRP) due to a significant HRP-independent activity of the carboxylesterase enzyme in the cortical mitochondria, which is inhibited by PMSF (Figure 5A and B). In addition, the carboxylesterase activity, which was indicated by the fluorescence slope after the addition of mitochondria in both -HRP and +HRP tests from male (Figure 5A) and female (Figure 5B) rats, was observed to be higher in the cortical mitochondria of females than males (Figure 5C). This carboxylesterase-induced conversion of AR to resorufin was dramatically reduced by the addition of PMSF and slightly reduced after the addition of succinate, without an apparent reason. Therefore, instead of a flat fluorescence after the addition of PMSF, a flat fluorescence after the addition of succinate was picked as the condition for optimizing the PMSF inhibition in the control (-HRP) runs. We also observed stabilization of the fluorescence signal with the addition of dimethyl sulfoxide (DMSO, 2 µL in 1 mL solution) (Figure 5A and B). Following inhibition of the carboxylesterase activity by PMSF, cortical mitochondrial H₂O₂ emission exhibited the expected pattern association with respiration: a high emission rate under succinate stimulation that decreased with ADP and FCCP additions, consistent with previous observations in heart and renal OM mitochondria.^40,41^ Succinate induced higher rates of H₂O₂ emission in cortical mitochondria compared to PM or GM (data not shown). Figure 5D-G shows the H₂O₂ emission at different respiration states. Despite the greater carboxylesterase activity observed in female cortical mitochondria, H₂O₂ emission rates during State 2 (basal respiration) and State 3 (ADP-stimulated respiration) were significantly lower in the cortical mitochondria of females compared to males (Figure 5D and E). No sex differences H₂O₂ emission were detected when respiration was completely uncoupled at State 5, which also has limited H₂O₂ emission (Figure 5F). Interestingly, the difference between State 2 and State 3 H₂O₂ emission, representing ADP-inhibitable H₂O₂ emission (Figure 5G), was significantly reduced in female cortical mitochondria. Figure 5J shows the representative H₂O₂ traces following the addition of mitochondria and succinate (10 mM) in a conventional protocol to measure reverse electron transport (RET) induced H₂O₂ emission. Mitochondria were pretreated with PMSF, and rotenone was added before the addition of succinate. The H₂O₂ emission rate in each group was quantified in Figure 5K. A significant reduction of H₂O₂ emission driven by succinate was observed in male mitochondria samples treated with rotenone. But no differences were observed in female mitochondria with and without rotenone. The rotenone-inhibitable H₂O₂ emission, ie, RET-driven H₂O₂ emission, was significantly higher in male mitochondria (Figure 5L).

**Figure 6.**
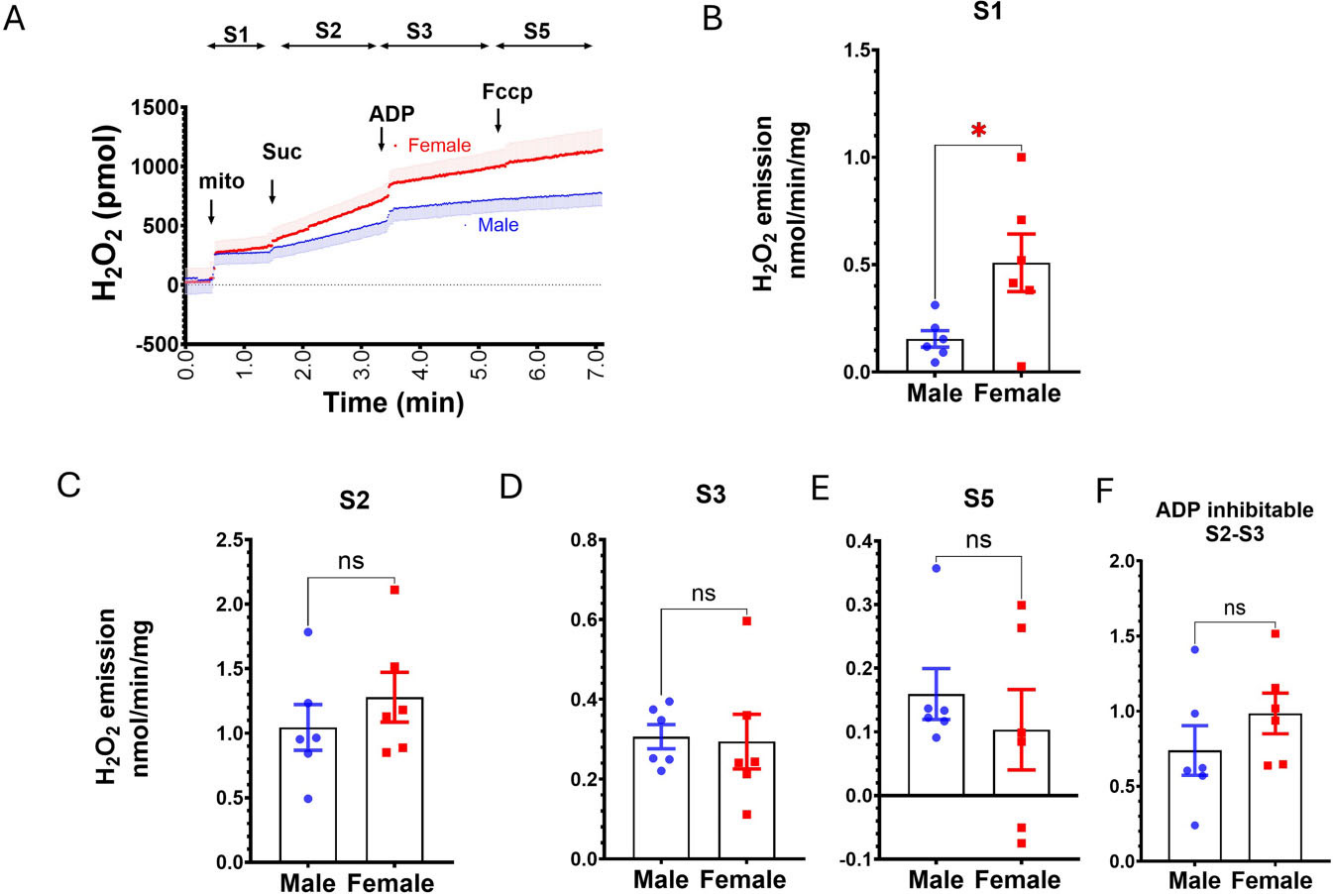
Renal OM mitochondrial H₂O₂ emission in male and female SS rats. A: Representative H_2_O_2_ emission trace during succinate-driven respiration in male (blue) and female (red) SS rats. B: Quantification of H₂O₂ emission at State 1, non-respiration associated signal. C-F Quantification of H₂O₂ emission at various respiratory states in OM mitochondria after correcting for non-mitochondrial contributions. Data are shown as mean ± SEM and individual values (*n* = 6 per group). Statistical significance was determined using a Student’s *t-*test. *P < 0.05

In OM mitochondria, a limited but detectable level of H₂O₂ emission at S1 was observed (Figure 6A and B). With limited OM mitochondria, the sources of S1 H₂O₂ emission have not been comprehensively studied, and their contribution to H₂O₂ emission at respiration states was considered by subtracting. Succinate induced a much higher rate of H₂O₂ emission compared to PM in OM mitochondria at State 2 respiration (data not shown). With the limited OM mitochondria, only succinate-driven H₂O₂ emission was compared between male and females. Interestingly, the H₂O₂ emission rates in OM mitochondria were higher in females than males cross all states at time course curves (Figure 6A) including at the State1 baseline without succinate (Figure 6B). However, after correcting to State 1 H₂O₂ emission, sex different H₂O₂ emissions at State 2, 3 and 5 respiration and ADP-inhibitable H₂O₂ emission were no longer evident (Figures 6C-F).

Direct comparisons of O_2_ consumption and H₂O₂ emission revealed a distinct tissue-specificity between the cortex and OM mitochondria (Supplemental Table S1). A two-way ANOVA showed a significantly higher O_2_ consumption in OM during both State 2 and State 3 respiration then in cortex, consistent with previous discovery.^46^ This was fueled by both PM, with consumption being 1.7 times higher in State 2 and 1.6 times higher in State 3, and succinate, which resulted in 1.3 times higher consumption in State 2 and 1.2 times higher in State 3. Even more striking was the disproportionately higher level of respiration-associated H₂O₂ emission from OM driven by succinate. This emission was a staggering 5.4 times higher in State 2 and 3.5 times higher in State 3 compared . The H₂O₂ emission that ADP could inhibit was 6.6 times higher in OM than in the cortex. Given the smaller size of the OM region to the whole kidney, the increase in O_2_ consumption might not impact overall kidney oxygenation. However, the substantial H₂O₂ emission from the OM is a factor that should not be overlooked.

### Mitochondrial Electron Transport Chain Complexes Abundance

**Figure 7.**
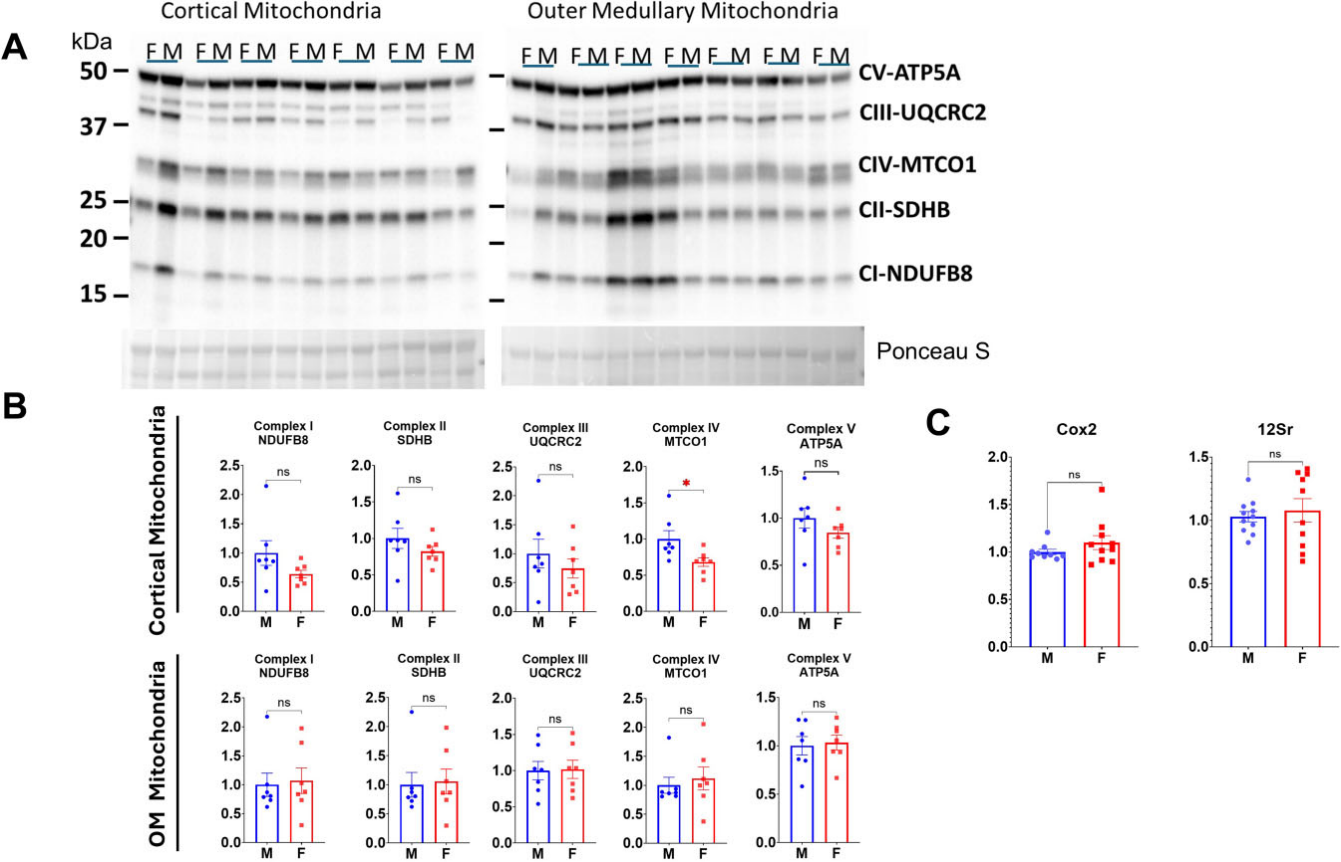
Sex-specific mitochondrial respiratory complex protein expression in isolated mitochondria from SS renal cortex and OM. (A) Western blots: OXPHOS complexes protein (ATP5A for complex V, UQCRC2 for complex III, MTCO1 for complex IV, SDHB for complex II, and NDUFB8 for complex I) in cortical and OM mitochondria, recognized by OXPHOS antibody cocktail. (B) Densitometric analysis: Comparison of each protein expression between male and female SS rats in mitochondria samples isolated from the cortex and OM. *n* = 7 per group. (C) Comparison of mitochondrial DNA copy number in cortical tissues of male and female SS rats, assessed from the mitochondrial genome site of the Cox2 gene (Cox2) and 12S ribosomal RNA gene (12Sr). *n* = 9-11 per group. Data are presented as mean ± SEM and individual values. Statistical significance was assessed using a Student’s *t*-test. *P < 0.05

Western blot analysis of isolated mitochondria from renal cortex and OM was performed using an OXPHOS antibody cocktail detecting representative subunits from ETC complexes I-V (Figure 7A). Protein densitometry was generally similar in cortical and OM mitochondria, indicating consistent protein loading across samples and a comparable mitochondrial purity between preparations. Given the higher mitochondrial isolation efficiency from the cortex compared to OM (Table 1), mitochondrial density at the cellular level was likely greater in the cortical tissue.

Quantitative Western blot analysis revealed that female cortical mitochondria exhibited a lower abundance of each representative ETC protein compared to males, with MTCO1 protein (mitochondrially encoded cytochrome c oxidase I) at complex IV showing a statistically significant reduction (Figure 7B), consistent with the observed lower cortical mitochondrial respiration and H_2_O_2_ emission in females (Figures 2, 3, and 5). In contrast, no sex-specific differences in ETC protein abundance were observed in OM mitochondria (Figure 7B), also consistent with the observed no sex differences in OM mitochondrial respiration or respiration-associated H_2_O_2_ emission in females and males (Figures 2, 4, and 6).

Examination of the relative protein composition within each sample (Figure 7A) revealed tissue-specific patterns of ETC complex abundance. ATP5A protein (ATP synthase lipid-binding protein) in complex V might be the most abundant subunit in both cortex and OM mitochondria. In cortex mitochondria, SDHB (succinate dehydrogenase complex iron-sulfur subunit) in complex II was the second most intense subunit, whereas UQCRC2 (ubiquinol-cytochrome c reductase core protein 2) in complex III was the second most intense in OM mitochondria, suggesting a relatively higher complex II activity in cortex and a greater complex III representation in OM relatively.

ETC protein expression was also evaluated in cortical and OM tissue homogenates (Figures 9A and 10A). The relative abundance profiles in tissue homogenates differed from those observed in isolated mitochondria. Notably, MTCO1 (complex IV) emerged as the second most intense protein in both cortical and OM tissue homogenates. These results indicated that MTCO1 protein expression was relatively high; however, its insertion into mitochondria did not reach the same extent as that of other proteins, suggesting a divergence between mitochondrial protein expression and mitochondrion particle assembly. Interestingly, ATP5A emerged as the most abundant protein in both cortical and OM tissue homogenates, similar to what was observed in isolated mitochondria. Despite these compositional differences, male cortical tissue homogenates displayed significantly higher MTCO1 abundance compared to females (Figure 9B). In contrast, females showed higher levels of most of the complex representative proteins in OM homogenates (Figure 10B). These results suggest that ETC protein expression had sex and tissue specificity, and elevated MTCO1 gene expression in the male renal cortex may contribute to the greater abundance of MTCO1 protein observed in isolated male cortical mitochondria, potentially enhancing respiration capacity in males of this region, and the mitochondrial function may be determined less closely by the gene expression in the cytosol.

**Figure 8.**
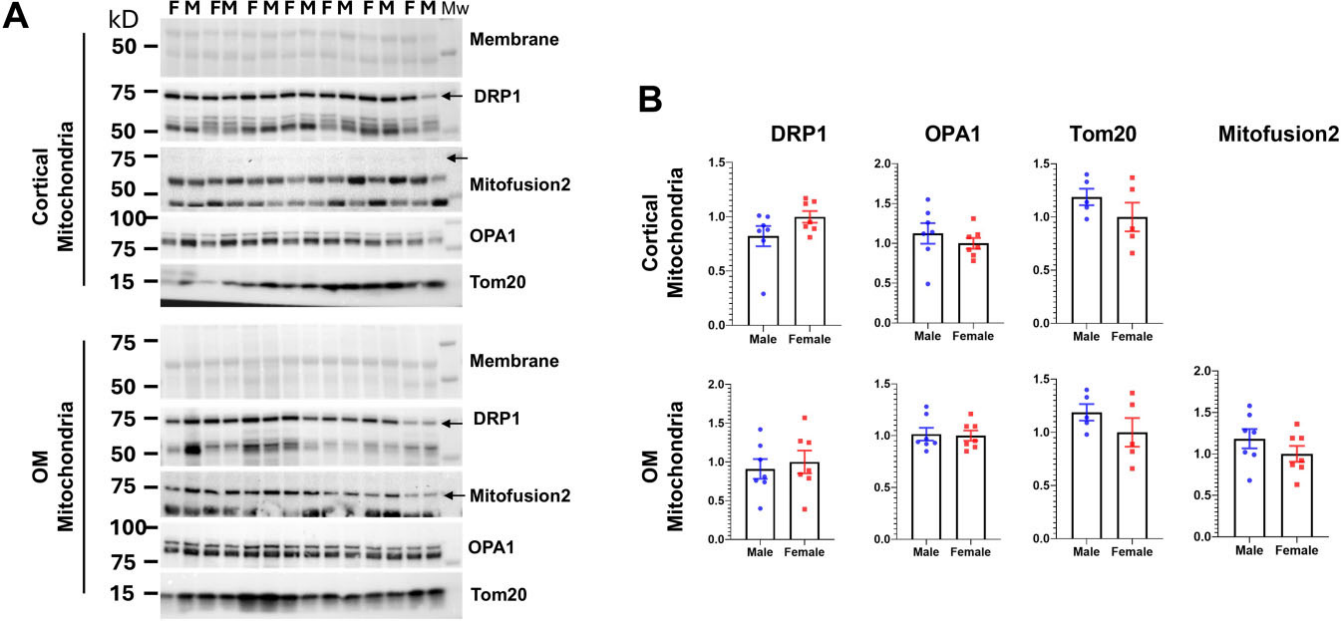
Western blot and quantitative analysis of mitochondrial fission and fusion proteins in isolated mitochondria from cortex and OM tissues. Panel A shows the Western blots themselves, which are images of the separated proteins. Panel B provides the quantitative analysis of these blots. A Student’s *t*-test was performed for each marker protein, but the analysis found no statistically significant difference between the groups for any of the proteins examined (*n* = 7). OPA1: Optic Atrophy 1; DRP1: Dynamin-related protein 1, Tom20: Translocase of Outer Mitochondrial Membrane 20.

Mitochondrial DNA (mtDNA) copy number was assessed in cortical tissues from both male and female rats (Figure 7C), which targeted two different sites on the mitochondrial genome, the Cox2 gene and the 12S ribosomal RNA gene, and produced identical results. There were no sex differences in the number of mtDNA copies relative to the nuclear genome. We also assessed potential differences in mitochondrial fission and fusion dynamics in males and females by measuring the abundance of key marker proteins in isolated mitochondria from both cortical and OM tissues, as shown in Figure 8. All tested antibodies successfully recognized unique proteins in the isolated mitochondrial samples. No sex-based differences were observed in the abundance of any of these proteins. The Mitofusin2 protein was found to be at a relatively lower abundance in cortical mitochondria compared to OM mitochondria, which precluded a reliable quantitative analysis for this protein in the cortical tissue.

### Sex-Specific Abundance of Transport Proteins Along the Nephron Segments of Dahl SS Rats

Previous studies quantified sex-specific expression of transporters and channels along the nephron segments of Sprague Dawley (SD) rats.^26,47^ To determine whether similar patterns exist in Dahl SS rats, Western blot analyses were performed to examine the abundance of the representative transporters identified in the SD rat study on the cortical and OM tissue homogenates from male and female SS rats.

**Figure 9.**
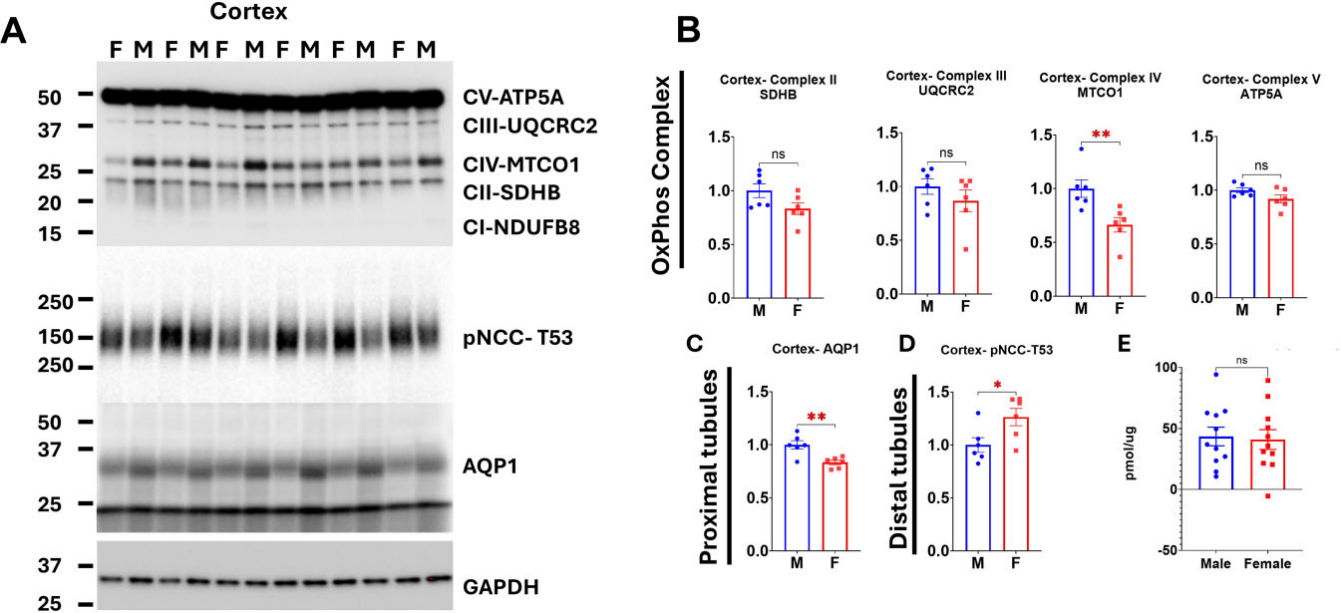
Sex-specific protein expression in renal cortex tissue homogenate. (A) Western blots: Representative OXPHOS complexes protein expression (ATP5A for complex V, UQCRC2 for complex III, MTCO1 for complex IV, SDHB for complex II, and NDUFB8 for complex I) and AQP1(aquaporin-1 water channel) are mainly expressed in proximal tubules, and pNCC-T53 (phosphorylated Na-Cl-cotransporter, NCC at threonine 53) is specific in distal tubules. (B-D): Densitometric analysis: Comparison of protein expression between male and female SS rats. (B) OXPHOS complexes protein expressions, (C) AQP1, and (D) p-NCC-T53. E: Comparison of cortical tissue Na-K ATPase activity between male and female SS rats. Data are presented as mean ± SEM and individual values; *n* = 6-11 per group. Statistical significance was assessed using a Student’s *t*-test. *P < 0.05. **P < 0.01.

Aquaporin 1 (AQP1), a key mediator of water reabsorption and sodium (Na⁺) transport coordination in the kidney PTs, was used as a marker for PTs Na⁺ reabsorption. Two AQP1 isoforms (∼35 kDa and ∼24 kDa) were detected in the cortical tissue homogenates (Figure 9A). The ∼35 kDa isoform was significantly less abundant in females compared to males (Figure 9C).

The Na⁺-Cl^−^ cotransporter (NCC), localized to the distal convoluted tubule (DCT). Its activated, phosphorylated form at threonine 53 (pNCC-T53) were detected in the cortical tissue homogenates (Figure 9A). In contrast to AQP1, pNCC-T53 were significantly more abundant in female SS rats (Figure 9D).

**Figure 10.**
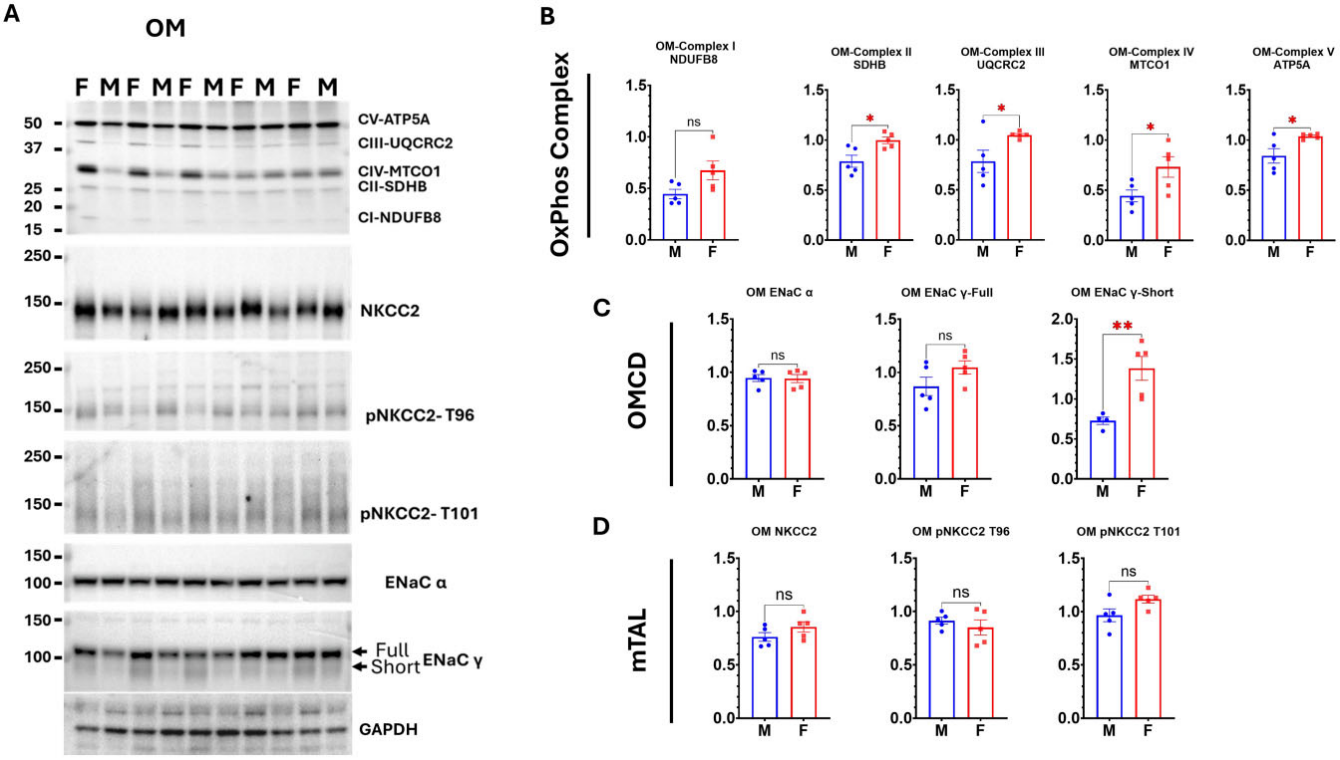
Sex-specific protein expression in renal OM tissue. (A) Western blots: Protein expression of mitochondrial respiratory complexes (ATP5A for complex V, UQCRC2 for complex III, MTCO1 for complex IV, SDHB for complex II, and NDUFB8 for complex I), sodium transporters (NKCC2, pNKCC2-T96, pNKCC2-T101), and epithelial sodium channel subunits (ENaC α, ENaC γ) in renal OM tissue homogenates. (B-D) Densitometric analysis: Comparison of protein expression levels between male and female SS rats. NKCC2: Na-K-2Cl cotransporter 2; pNKCC2-T96: Phosphorylated NKCC2 at threonine 96; pNKCC2-T101: Phosphorylated NKCC2 at threonine 101; ENaC: Epithelial sodium channel; mTAL: Medullary thick ascending limb of Henle’s loop; OMCD: OM collecting duct. Data are presented as mean ± SEM and individual values; *n* = 5 per group. Statistical significance was determined using a Student’s *t*-test. *P < 0.05; **P < 0.01.

In the OM, Na^+^ reabsorption downstream of the PT occurs predominantly in the medullary thick ascending limb (mTAL) via the Na⁺-K⁺-2Cl^−^ cotransporter isoform 2 (NKCC2). Western blot analysis of the OM tissue homogenates identified total NKCC2 protein and two phosphorylated, activated isoforms (pNKCC2-T96 and pNKCC2-T101) (Figure 10A). Consistent with observations in SD rats, a close to significant trend toward higher pNKCC2-T101 levels was observed in female SS rats (Figure 10C).

The epithelial Na⁺ channel (ENaC), composed of α, β, and γ subunits, is expressed along the late DCT, connecting tubule, and collecting duct in both cortex and OM. The α and γ subunits serve as markers of apical membrane channel activation.^44,48^ Total ENaC α, as well as both full-length and cleaved isoforms of ENaC γ, were detected in the OM tissue homogenates. Notably, the cleaved (short) isoform of ENaC γ was significantly more abundant in females (Figure 10D).

Ouabain-inhibited Na-K-ATPase activity was measured in membrane fractions from cortical tissue. As shown in Figure 9E, no sex differences were detected in this activity.

## Discussion

This study investigated the sex- and tissue-specific differences in respiratory capacity and H_2_O_2_ emission in isolated kidney mitochondria from young male and female Dahl SS rats. The mitochondria were isolated from the renal cortex and OM. We used the compound PMSF to reduce background fluorescence in AR + HRP assay, allowing for more precise quantification of H_2_O_2_ emission under various respiratory conditions. Contrary to the common belief that females have superior respiratory capacity, our results indicate that the kidneys of male SS rats possess a higher respiratory capacity and emit more H_2_O_2_. We also briefly explored the plausible reasons for these surprising sex-specific differences.

### Sexual Dimorphism in Mitochondrial Bioenergetic Output in the SS Rat Kidney

We hypothesized that, due to the maternal inheritance of mitochondria and the known effects of estrogen on mitochondrial respiration,^7,17^ sex differences in kidney mitochondrial respiratory function would parallel those reported in other organs, where females often exhibit higher activity. Contrary to this prediction, the present study found marked sex- and region-specific differences in the kinetics of mitochondrial oxidative phosphorylation (OXPHOS) capacity in the kidneys of young Dahl SS rats on a low-salt diet.

In the renal cortex, males displayed significantly higher ADP-stimulated (State 3) O₂ consumption than females, indicating a greater intrinsic capacity for mitochondrial ATP production. In contrast, mitochondria isolated from the renal OM showed no sex-specific differences in the kinetics of OXPHOS, suggesting that mitochondrial bioenergetics are differentially regulated along the corticomedullary axis.

In both cortical and OM mitochondria, the FADH₂-linked substrate succinate elicited higher State 3 respiration than NADH-linked substrates (pyruvate + malate or glutamate + malate), consistent with prior observations in other tissues.^39-41^ The sex- and tissue-specific differences in cortical JO₂ were evident across substrates, with males exhibiting not only higher State 3 respiration but also trends toward elevated State 2 and State 4 respiration for NADH-linked substrates (Figures 2, 3). These findings implicate enhanced electron transport chain (ETC) activity as a key driver of increased OXPHOS in male cortical mitochondria.

Mitochondrial respiratory function could be significantly influenced by factors such as mitochondrial DNA (mtDNA) copy number ^49^ and the dynamic processes of fission and fusion.^7^ To investigate the basis of the observed sex-specific difference in respiratory capacity, we examined these parameters. Despite functional disparities, our analysis revealed no significant sex differences in either the mtDNA copy number in frozen cortex tissues (Figure 7C) or in the imbalance of fission and fusion in isolated mitochondria (Figure 8).

Western blot analysis on ETC proteins revealed significantly greater abundance of the Complex IV subunit MTCO1 in male cortical mitochondria, with no corresponding differences in OM mitochondria. Elevated MTCO1 was also observed in male cortical tissue homogenates, indicating a difference in gene expression. This functional enhancement of respiration was coupled with markedly greater H₂O₂ emission during State 3 respiration in male cortical mitochondria (Figure 5G), linking increased forward electron flux to greater oxidative byproduct generation. Our findings indicate that a higher mitochondrial respiratory function in male renal cortical tissues is likely driven by increased MTCO1 gene expression in the host cells. However, further investigation is needed to determine whether this enhancement is due solely to the MTCO1 subunit, which is uniquely encoded by the mitochondrial genome, or to broader changes in complex IV compositions that contribute to elevated ETC activity in the male cortical mitochondria.

Our OXPHOS analysis protocol closely modeled in vivo respiration which typically occurs between State 3 and State 4.^47^ Thus, the higher State 3 JO₂ in male cortical mitochondria, alongside similar OXPHOS coupling (P/O ratios) between sexes (Figure 3J, P), suggests a genuine increase in ATP synthesis capacity in males. Although ATP production was not measured directly in vivo, our data predicted an intrinsically greater cortical mitochondrial bioenergetic output in male SS rats, and a higher ATP-generating capacity per PT, given the substantial PT mass in the cortical region.

Based on our OM mitochondria respiratory data, OM tissue O_2_ consumption might be the same between male and female SS rats. However, due to the complex cellular composition and functional heterogeneity within this region, it is not possible to determine whether O_2_ consumption is equivalent across individual cell types.

### Mitochondrial H₂O₂ Emission and Sex Differences in SS Rats

Oxidative stress is a key contributor to renal injury and a well-established driver of SS hypertension in both SS rats and humans.^36-38^ Among mitochondrial-derived ROS, H₂O₂ serves as a critical byproduct of ETC activity and is widely used as a marker of mitochondrial redox status.

In this study, we optimized the use of AR/HRP assay to accurately quantify mitochondrial H_2_O_2_ emission associated with ETC activity in cortical mitochondria. In our previous works,^40,41^ we observed that the traditional AR/HRP assay failed to detect changes in H_2_O_2_ emission rate in response to altered respiration. Miwa et al.^43^ reported a significant HRP-independent fluorescence in liver mitochondria, which can be suppressed by PMSF, a serine protease/esterase inhibitor. This was attributed to carboxylesterase activity consistent with their detection of carboxylesterase proteins in rat liver and kidney. RNAseq data revealed that carboxylesterases gene expression is predominantly localized to PT segments of the nephron, with expression levels approximately 30-fold lower in mTAL/OMCD and other OM segments.^50^

We confirmed that PMSF significantly suppressed HRP-independent fluorescence, allowing for the accurate measurement of respiration-associated H_2_O_2_ emission in renal cortical mitochondria. PMSF dosing required careful optimization, as excessive concentrations reduced signal following succinate addition. Notably, PMSF dose requirements differed by sex: female cortical mitochondria exhibiting higher baseline background fluorescence, necessitating individualized titration. Due to PMSF’s broad reactivity and dose sensitivity, reliable quantification of State 1 (non-respiratory) H_2_O_2_ emission in cortical samples was not feasible. Control runs (-HRP) for cortical mitochondria were set to ensure that the succinate induced zero fluorescence change.

Carboxylesterase activity appears to be quite low in OM mitochondria. Our limited data from male OM mitochondria in control (-HRP) run showed flat fluorescence signal while an increasing fluorescence signal was detected from assay (+HRP) run, indicating a State 1 H_2_O_2_ emission from non-respiratory reactions (data not shown). The higher State 1 H_2_O_2_ emission in female OM mitochondria (Figure 6B) was not comprehensively addressed in this study. OM mitochondrial H_2_O_2_ emission associated with respiration was calculated after subtracting the State 1 H_2_O_2_ emission.

Using this optimized approach, we found that succinate induced the highest H₂O₂ emission rates in both cortical and OM mitochondria (PM, GM data not shown), a pattern consistent with findings in other tissues and species.^40,41,51,52^ However, a striking regional distinction emerged: OM mitochondria exhibited approximately five-fold more H₂O₂ emission than cortical mitochondria during succinate-driven State 2 respiration and three-fold higher during State 3 respiration. These data identify the OM as a significant “hotspot” for mitochondrial ROS generation in the SS rat kidney, consistent with prior studies.^37^

We quantified two components of succinate-driven H₂O₂ emission using our OXPHOS protocol: (1) State 3 H₂O₂ emission, reflecting ROS generation during active ATP synthesis, and (2) ADP-inhibitable H₂O₂ emission, calculated as the difference between State 2 and State 3, reflecting H₂O₂ emission suppressed by ADP phosphorylation, potentially due to reverse electron transport (RET). Sex differences in ADP-inhibitable H₂O₂ emission in cortical mitochondria aligned with higher rotenone-inhibitable H₂O₂ emission (a conventional measurement of RET) in males, supporting the interpretation that ADP-inhibitable H₂O₂ emission reflects RET-derived ROS production. Although a sex difference was evident by this conventional protocol, there was no significant difference in females with and without rotenone. This may be due to the low level of H₂O₂ emission and limited sensitivity of the assay at these levels. Variability in PMSF-mediated background fluorescence suppression may also contribute to reduced sensitivity.

While RET accounts for a substantial portion of mitochondrial H₂O₂ generation under certain conditions, State 3 H₂O₂ emission measured in the study is likely more physiologically relevant, as it reflects mitochondrial function during ATP synthesis when mitochondrial antioxidant systems remain active. The values we measured reflect the equilibrium level of H₂O₂, rather than its total production. Therefore, we refer to our measurement as H₂O₂ emission to accurately represent the results from the balance between production and antioxidant-mediated clearance. In male cortical mitochondria State 3 H₂O₂ emission was significantly higher than in females, consistent with elevated respiration and ADP turnover. These sex differences in respiration-linked H₂O₂ emission were not observed in OM mitochondria, where overall emission was higher but not sex dependent.

Collectively, these findings suggest that male cortical mitochondria not only exhibit greater OXPHOS capacity but also produce more ROS during normal respiratory activity. This coupling of energy production and redox imbalance appears to contribute to increased oxidative stress in the renal cortex of male SS rats. Over time, sustained ROS accumulation, particularly H₂O₂, would be expected to lead to cellular damage and compromise tubular function.^53,54^ This mechanism may underlie the observed vulnerability of male SS rats to renal injury even under low-salt conditions ^55^ and further explains the sex-specific progression of kidney injury and dysfunction during high-salt dietary challenges. Our findings position mitochondrial redox imbalance as a mechanistic link between nephron workload, bioenergetics, and sex-specific susceptibility to renal pathophysiology in SS hypertension.

### Connecting Mitochondrial Bioenergetics to Renal Tubular Function

Our OXPHOS analysis indicates enhanced mitochondrial respiratory activity in the renal cortex of male SS rats. Given that the renal cortex is composed predominantly of PTs, this finding likely reflects the elevated ATP production to match a higher ATP demand to support sodium reabsorption in these segments. The PT is responsible for reclaiming the majority of filtered solutes and water, and these transport processes are highly energy-dependent, relying almost exclusively on mitochondrial oxidative metabolism.^53-56^ Previous work, notably by McDonough and colleagues,^22,47,57^ has shown that female rodents exhibit lower fractional sodium and water reabsorption in the PT, accompanied by a compensatory increase in distal nephron transport, a phenomenon referred to as a “downstream shift” in sodium reabsorption.

Our protein expression data in SS rats support this model. In cortical tissue homogenates from male SS rats, we observed increased expression of aquaporin-1 (AQP1), a water channel highly expressed in the PT and positively associated with sodium reabsorption.^30^ Conversely, phosphorylated NCC at threonine 53 (pNCC T53), a marker of distal tubule sodium reabsorption,^44,48^ was reduced in males. In OM tissue, we detected a greater abundance of the γ-short isoform of epithelial sodium channel (ENaC) in female rats. This splice variant is linked to higher ENaC activity and sodium reabsorption in distal segments.^58-61^ Together, these data reinforce the notion that females shift a greater proportion of sodium transport to distal nephron segments. At the same time, males rely more heavily on proximal reabsorption, imposing a correspondingly higher bioenergetic burden on male cortical mitochondria.

This sex-specific correspondence between mitochondrial ATP production and tubular transport workload in the cortical region highlights a tight coupling between mitochondrial ATP production capacity and their host epithelial cell ATP demand in the PT segment. In PT cells, where glycolytic ATP production is minimal, OXPHOS must meet nearly all of the local energy demands in physiological conditions.^55^ Thus, the enhanced mitochondrial bioenergetic output observed in male cortical mitochondria appears to directly support increased PT sodium transport, likely overriding the patterns typically associated with sex-dependent mitochondrial function, which are often influenced by maternal inheritance and sex hormones. This “functional override” of mitochondrial OXPHOS may be a necessary evolutionary adaptation to preserve the PT’s low glycolytic phenotype while sustaining its high metabolic workload. Supporting this, increased glycolytic flux has been observed in the PT during various renal injury states, representing a pathological shift from this evolutionarily conserved metabolic profile.^62^

Interestingly, we did not detect significant sex differences in mitochondrial respiration or H₂O₂ emission in the OM, despite robust sex differences in protein markers of distal transport. This absence of a difference cannot be attributed solely to the heterogeneity of cell types in this region, because if maternal inheritance and the proposed downstream shift were universally dominant, one might expect a greater proportion of cells to favor higher OXPHOS in females. The slightly lower, though non-significant, respiratory control index (RCI) observed in female OM mitochondria with both complex I- and complex II-linked substrates may reflect lesser female mitochondrial integrity. Thus, both respiration capacity and H₂O₂ emission were reduced to the same level as those of males. Hormonal status of the female rats was not controlled in this study, and the use of females from the same litters, tested over a 2-3-week period, likely included animals at different stages of the estrous cycle. However, in each experiment, females paired with males consistently exhibited lower cortical mitochondrial respiration and H₂O₂ emission, effectively excluding hormonal status as the primary explanation for our findings.

### Sex Dimorphic Renal Injury and Salt Sensitivity in SS Rats: The Role of Mitochondrial Function and ROS Production

This study reveals sex-specific differences in mitochondrial function that correspond with known sex dimorphisms in renal physiology and susceptibility to injury in SS rats. In males, elevated mitochondrial respiratory activity in the renal cortex is coupled with increased production of superoxide (O_2_^−^) and its downstream product, H₂O₂, byproducts of mitochondrial OXPHOS that play dual roles in cellular signaling and redox stress. While low levels of mitochondrial ROS contribute to physiological signaling, their sustained overproduction can lead to oxidative stress. This stress can damage cellular components, such as proteins, lipids, and DNA, and alter gene expression.

Our findings suggest that the elevated O_2_^−^/H_2_O_2_ production observed in male cortical mitochondria may contribute to a progressive redox imbalance chronically, rendering the male renal cortex more vulnerable to injury. This is consistent with previous reports documenting increased renal damage in male SS rats as early as six months of age, even under normal salt intake conditions.^11^

The connection of mitochondrial OXPHOS capacity and renal tubular function strongly suggests that the mitochondria would be involved in the progression of salt-induced hypertension. Increased sodium reabsorption in the PT, driven by HS intake, imposes additional ATP demand, amplifying mitochondrial respiration and ROS production. This cascade likely contributes to salt-induced renal dysfunction and hypertension, as repeatedly demonstrated in male SS rats exposed to HS diets in our laboratory.^14,15,38^

The OM is another site of particular vulnerability in SS rats. We observed markedly higher H₂O₂ emission from OM mitochondria compared to cortical mitochondria, regardless of sex. Given the lower oxygen tension and high transport activity in this region, these findings support prior studies implicating medullary oxidative stress as a central contributor to salt-induced hypertension in the SS rat.^38,39,63,64^ While sex differences in OM mitochondrial H_2_O_2_ emission were not observed under low-salt conditions, the high baseline redox activity suggests that the OM may be especially susceptible to oxidative damage when salt intake is high.

## Summary and Conclusions

This study identifies sex-specific differences in renal mitochondrial bioenergetics in Dahl SS rats. Male cortical mitochondria exhibited elevated O_2_ consumption, ATP production, H_2_O_2_ emission, and Complex IV protein abundance, consistent with higher PT transport demands, suggesting increased susceptibility of the cortical region to oxidative injury. In contrast, females exhibited lower cortical mitochondrial respiratory activity and a downstream shift in tubular transport, which may protect against cortical oxidative damage. These findings underscore the importance of sex as a biological variable in renal metabolism and disease.

### Limitations of the Study and Future Directions

This study assessed mitochondrial respiration and H_2_O_2_ emission in the kidney cortex and OM using in vitro isolated mitochondrial preparations, which, while informative, do not fully capture in vivo mitochondrial function, including O_2_ consumption, ATP production, and H₂O₂ emission under physiological conditions. The “artificial” saturated substrate concentrations used in the protocols could also not be achieved in physiological conditions. Future studies employing in vivo approaches such as mitochondrial fluxomics and metabolomics are needed to validate and extend these findings. Additionally, intact isolated PTs and medullary thick ascending limbs (mTAL) can be used in the high-throughput Seahorse system under physiological circulating substrate concentrations to study sex differences in mitochondrial function in these segments, rather than isolated mitochondria from the cortical and OM tissues. Finally, lower tissue and mitochondrial yield from the OM limited the depth of analysis in this region, warranting further investigation and innovation in methodology, such as optimizing and using the OM tissue homogenate in the Seahorse system.

## Supplementary Material

zqaf045_Supplemental_File

## Data Availability

The data that support the findings of this study are available from the corresponding author upon reasonable request.

## References

[1] Mauvais-Jarvis F, Bairey Merz N, Barnes PJ et al. Sex and gender: modifiers of health, disease, and medicine. The Lancet. 2020;396(10250):565–582. 10.1016/S0140-6736(20)31561-0 PMC7440877 32828189

[2] Wilkinson NM, Chen HC, Lechner MG, Su MA. Sex differences in immunity. Annu. Rev. Immunol. 2022;40:(1):75–94. 34985929 10.1146/annurev-immunol-101320-125133 PMC9805670

[3] Costanzo M, Caterino M, Sotgiu G, Ruoppolo M, Franconi F, Campesi I. Sex differences in the human metabolome. Biol Sex Differ. 2022;13(1):30. 35706042 10.1186/s13293-022-00440-4 PMC9199320

[4] Clotet-Freixas S, Zaslaver O, Kotlyar M et al. Sex differences in kidney metabolism may reflect sex-dependent outcomes in human diabetic kidney disease. Sci. Transl. Med. 2024;16(737):eabm2090. 38446901 10.1126/scitranslmed.abm2090

[5] Colafella KMM, Denton KM. Sex-specific differences in hypertension and associated cardiovascular disease. Nat Rev Nephrol. 2018;14(3):185–201. 29380817 10.1038/nrneph.2017.189

[6] Arnold AP, Cassis LA, Eghbali M, Reue K, Sandberg K. Sex hormones and Sex chromosomes cause Sex differences in the development of cardiovascular diseases. ATVB. 2017;37(5):746–756. 10.1161/ATVBAHA.116.307301 PMC5437981 28279969

[7] Ventura-Clapier R, Moulin M, Piquereau J, Mitochondria: a central target for sex differences in pathologies. Clin Sci (Lond). 2017;131(9):803–822. 28424375 10.1042/CS20160485

[8] McCrimmon A, Cahill KM, Kruger C et al. Intact mitochondrial substrate efflux is essential for prevention of tubular injury in a sex-dependent manner. JCI Insight. 2022;7(7):1–24. 10.1172/jci.insight.150696 PMC9057616 35230975

[9] Rudemiller NP, Mattson DL. Candidate genes for hypertension: insights from the Dahl S rat. Am J Physiol Renal Physiol. 2015;309(12):F993–F995. 25877508 10.1152/ajprenal.00092.2015 PMC4839476

[10] Wang Z, Sun Q, Sun N, Liang M, Tian Z. Mitochondrial dysfunction and altered renal metabolism in Dahl salt-sensitive rats. Kidney Blood Press Res. 2017;42(3):587–597. 28922660 10.1159/000479846

[11] Turbeville HR, Johnson AC, Garrett MR, Dent EL, Sasser JM. Nitric oxide and oxidative stress pathways do not contribute to sex differences in renal injury and function in Dahl SS/Jr rats. Physiol Rep. 2020;8(13):e14440. 32652814 10.14814/phy2.14440 PMC7354091

[12] Gong G, Dobin A, Johnson ML, Pettinger WA. Sexual dimorphism of renal alpha2-adrenergic receptor regulation in Dahl rats. Hypertens Res. 1996;19(2):83–89. 10968200 10.1291/hypres.19.83

[13] Fehrenbach DJ, Abais-Battad JM, Dasinger JH et al. Sexual dimorphic role of CD14 (Cluster of Differentiation 14) in salt-sensitive hypertension and renal injury. Hypertension. 2021;77(1):228–240. 33249861 10.1161/HYPERTENSIONAHA.120.14928 PMC9732708

[14] Abais-Battad JM, Dasinger JH, Lund H, Sex-dependency of T cell-induced salt-sensitive hypertension and kidney damage. Hypertension. 2024;81(7):1511–1523. 38757269 10.1161/HYPERTENSIONAHA.123.22608 PMC11168867

[15] Kim K, Nist KM, Puleo F, McKenna J 3rd, Wainford RD. Sex differences in dietary sodium evoked NCC regulation and blood pressure in male and female Sprague-Dawley, Dahl salt-resistant, and Dahl salt-sensitive rats. Am J Physiol Renal Physiol. 2024;327(2):F277–F289. 38813592 10.1152/ajprenal.00150.2023 PMC11460337

[16] Fernandes R, Garver H, Harkema JR, Galligan JJ, Fink GD, Xu H. Sex differences in renal inflammation and injury in high-fat diet-fed Dahl salt-sensitive rats. Hypertension. 2018;72(5):e43–e52. 30354819 10.1161/HYPERTENSIONAHA.118.11485 PMC6207243

[17] Sultanova RF, Schibalski R, Yankelevich IA, Stadler K, Ilatovskaya DV. Sex differences in renal mitochondrial function: a hormone-gous opportunity for research. Am J Physiol Renal Physiol. 2020;319(6):F1117–F1124. 33135479 10.1152/ajprenal.00320.2020 PMC7792688

[18] Ransick A, Lindström NO, Liu J, Single-cell profiling reveals sex, lineage, and regional diversity in the mouse kidney. Dev Cell. 2019;51(3):399–413.e7. 31689386 10.1016/j.devcel.2019.10.005 PMC6948019

[19] Huang L, Liao J, He J, Single-cell profiling reveals sex diversity in human renal proximal tubules. Gene. 2020;752:144790. 32439376 10.1016/j.gene.2020.144790

[20] Sabolić I, Asif AR, Budach WE, Wanke C, Bahn A, Burckhardt G. Gender differences in kidney function. Pflugers Arch. 2007;455(3):397–429. 17638010 10.1007/s00424-007-0308-1

[21] Chen L, Chou CL, Yang CR, Knepper MA. Multiomics analyses reveal sex differences in mouse renal proximal subsegments. JASN. 2023;34(5):829–845. 36758122 10.1681/ASN.0000000000000089 PMC10125651

[22] McDonough AA, Harris AN, Xiong LI, Layton AT. Sex differences in renal transporters: assessment and functional consequences. Nat Rev Nephrol. 2024;20(1):21–36. 37684523 10.1038/s41581-023-00757-2 PMC11090267

[23] Hu R, McDonough AA, Layton AT. Sex differences in solute and water handling in the human kidney: modeling and functional implications. iScience. 2021;24(6):102667. 34169242 10.1016/j.isci.2021.102667 PMC8209279

[24] Hu R, McDonough AA, Layton AT. Sex differences in solute transport along the nephrons: effects of Na(+) transport inhibition. Am J Physiol Renal Physiol. 2020;319(3):F487–F505. 32744084 10.1152/ajprenal.00240.2020 PMC7509281

[25] McDonough AA, Layton AT. Sex differences in renal electrolyte transport. Curr Opin Nephrol Hypertens. 2023;32(5):467–475. 37382185 10.1097/MNH.0000000000000909 PMC10526720

[26] Hu R, McDonough AA, Layton AT. Functional implications of the sex differences in transporter abundance along the rat nephron: modeling and analysis. Am J Physiol Renal Physiol. 2019;317(6):F1462–F1474. 31566436 10.1152/ajprenal.00352.2019 PMC7132321

[27] Hosszu A, Fekete A, Szabo AJ. Sex differences in renal ischemia-reperfusion injury. Am J Physiol Renal Physiol. 2020;319(2):F149–F154. 32567347 10.1152/ajprenal.00099.2020

[28] Hinojosa-Laborde C, Lange DL, Haywood JR. Role of female sex hormones in the development and reversal of dahl hypertension. Hypertension. 2000;35(1):484–489. 10642346 10.1161/01.hyp.35.1.484

[29] Wen Y, Qi H, Østergaard Mariager C, Sex differences in kidney function and metabolism assessed using hyperpolarized [1-(13)C]pyruvate interleaved spectroscopy and nonspecific imaging. Tomography. 2020;6(1):5–13. 32280745 10.18383/j.tom.2020.00022 PMC7138520

[30] Li Q, McDonough AA, Layton HE, Layton AT. Functional implications of sexual dimorphism of transporter patterns along the rat proximal tubule: modeling and analysis. Am J Physiol Renal Physiol. 2018;315(3):F692–F700. 29846110 10.1152/ajprenal.00171.2018 PMC6172582

[31] Thomsen K, Shirley DG. The validity of lithium clearance as an index of sodium and water delivery from the proximal tubules. Nephron. 1997;77(2):125–138. 9346378 10.1159/000190264

[32] Liu J, Yan Y, Liu L. Impairment of Na/K-ATPase signaling in renal proximal tubule contributes to Dahl salt-sensitive hypertension. J Biol Chem. 2011;286(26):22806–22813. 21555512 10.1074/jbc.M111.246249 PMC3123048

[33] Yang LE, Sandberg MB, Can AD, Pihakaski-Maunsbach K, McDonough AA. Effects of dietary salt on renal Na+ transporter subcellular distribution, abundance, and phosphorylation status. Am J Physiol Renal Physiol. 2008;295(4):F1003–F1016. 18653479 10.1152/ajprenal.90235.2008 PMC2576159

[34] Hoagland KM, Flasch AK, Dahly-Vernon AJ, dos Santos EA, Knepper MA, Roman RJ. Elevated BSC-1 and ROMK expression in Dahl salt-sensitive rat kidneys. Hypertension. 2004;43(4):860–865. 14967839 10.1161/01.HYP.0000120123.44945.47

[35] Alvarez-Guerra M, Garay RP. Renal Na-K-Cl cotransporter NKCC2 in Dahl salt-sensitive rats. J Hypertens. 2002;20(4):721–727. 11910309 10.1097/00004872-200204000-00031

[36] Cowley AW Jr., Roman RJ, Mattson DL et al. Renal Medulla in hypertension. Hypertension. 2024;81(12):2383–2394. 39344517 10.1161/HYPERTENSIONAHA.124.21711 PMC11578791

[37] Zou AP, Li N, Cowley AW Jr. Production and actions of superoxide in the renal medulla. Hypertension. 2001;37(2):547–553. 11230333 10.1161/01.hyp.37.2.547

[38] Cowley AW Jr . Renal medullary oxidative stress, pressure-natriuresis, and hypertension. Hypertension. 2008;52(5):777–786. 18852392 10.1161/HYPERTENSIONAHA.107.092858 PMC2659638

[39] Kuczeriszka M, Wąsowicz K. Animal models of hypertension: the status of nitric oxide and oxidative stress and the role of the renal medulla. Nitric Oxide. 2022;125-126:40–46. 35700961 10.1016/j.niox.2022.06.003

[40] Tomar N, Zhang X, Kandel SM, Substrate-dependent differential regulation of mitochondrial bioenergetics in the heart and kidney cortex and outer medulla. Biochimica et Biophysica Acta (BBA) Bioenergetics. 2022;1863(2):148518. 34864090 10.1016/j.bbabio.2021.148518 PMC8957717

[41] Sadri S, Tomar N, Yang C, Audi SH, Cowley AW Jr., Dash RK. Effects of ROS pathway inhibitors and NADH and FADH(2) linked substrates on mitochondrial bioenergetics and ROS emission in the heart and kidney cortex and outer medulla. Arch Biochem Biophys. 2023;744(8):109690. 37429534 10.1016/j.abb.2023.109690 PMC10528392

[42] Zhang X, Tomar N, Kandel SM, Audi SH, Cowley AW Jr., Dash RK. Substrate- and calcium-dependent differential regulation of mitochondrial oxidative phosphorylation and energy production in the heart and kidney. Cells. 2021;11(1):131. 35011693 10.3390/cells11010131 PMC8750792

[43] Miwa S, Treumann A, Bell A, Carboxylesterase converts amplex red to resorufin: implications for mitochondrial H2O2 release assays. Free Radical Biol Med. 2016;90:173–183. 26577176 10.1016/j.freeradbiomed.2015.11.011 PMC4708625

[44] Yang C, Isaeva E, Shimada S, Inhibition of mTORC2 promotes natriuresis in Dahl salt-sensitive rats via the decrease of NCC and ENaC activity. Am J Physiol Renal Physiol. 2024;327(3):F435–F449. 38779754 10.1152/ajprenal.00403.2023 PMC11460535

[45] Rutkai I, Dutta S, Katakam PV, Busija DW. Dynamics of enhanced mitochondrial respiration in female compared with male rat cerebral arteries. Am J Physiol Heart Circulat Physiol. 2015;309(9):H1490–H1500. 10.1152/ajpheart.00231.2015 PMC4666975 26276815

[46] Schiffer TA, Gustafsson H, Palm F. Kidney outer medulla mitochondria are more efficient compared with cortex mitochondria as a strategy to sustain ATP production in a suboptimal environment. Am J Physiol Renal Physiol. 2018;315(3):F677–F681. 29846107 10.1152/ajprenal.00207.2018

[47] Veiras LC, Girardi ACC, Curry J et al. Sexual dimorphic pattern of renal transporters and electrolyte homeostasis. JASN. 2017;28(12):3504–3517. 28774999 10.1681/ASN.2017030295 PMC5698077

[48] Vallon V, Schroth J, Lang F, Kuhl D, Uchida S. Expression and phosphorylation of the Na+-Cl- cotransporter NCC in vivo is regulated by dietary salt, potassium, and SGK1. Am J Physiol Renal Physiol. 2009;297(3):F704–F712. 19570885 10.1152/ajprenal.00030.2009 PMC2739704

[49] Qian W, Van Houten B. Alterations in bioenergetics due to changes in mitochondrial DNA copy number. Methods. 2010;51(4):452–457. 20347038 10.1016/j.ymeth.2010.03.006

[50] Lee JW, Chou CL, Knepper MA. Deep sequencing in microdissected renal tubules identifies Nephron segment-specific transcriptomes. J Am Soc Nephrol. 2015;26(11):2669–2677. 25817355 10.1681/ASN.2014111067 PMC4625681

[51] Wong HS, Dighe PA, Mezera V, Monternier PA, Brand MD. Production of superoxide and hydrogen peroxide from specific mitochondrial sites under different bioenergetic conditions. J Biol Chem. 2017;292(41):16804–16809. 28842493 10.1074/jbc.R117.789271 PMC5641882

[52] Brand MD . Riding the tiger—physiological and pathological effects of superoxide and hydrogen peroxide generated in the mitochondrial matrix. Crit Rev Biochem Mol Biol. 2020;55(6):592–661. 33148057 10.1080/10409238.2020.1828258

[53] Klein KL, Wang MS, Torikai S, Davidson WD, Kurokawa K. Substrate oxidation by isolated single nephron segments of the rat. Kidney Int. 1981;20(1):29–35. 7300110 10.1038/ki.1981.100

[54] Wirthensohn G, Guder WG. Renal substrate metabolism. Physiol Rev. 1986;66(2):469–497. 2938198 10.1152/physrev.1986.66.2.469

[55] Tian Z, Liang M. Renal metabolism and hypertension. Nat Commun. 2021;12(1):963. 33574248 10.1038/s41467-021-21301-5 PMC7878744

[56] Kanasaki K . The aberrant glycolysis in kidney proximal tubule: potential therapeutic target for DKD. Kidney Int. 2023;104(6):1056–1059. 37981426 10.1016/j.kint.2023.09.019

[57] Torres-Pinzon DL, Ralph DL, Veiras LC, McDonough AA. Sex-specific adaptations to high-salt diet preserve electrolyte homeostasis with distinct sodium transporter profiles. Am J Physiol Cell Physiol. 2021;321(5):C897–C909. 34613843 10.1152/ajpcell.00282.2021 PMC8616593

[58] Kleyman TR, Eaton DC. Regulating ENaC’s gate. Am J Physiol Cell Physiol. 2020;318(1):C150–C162. 31721612 10.1152/ajpcell.00418.2019 PMC6985836

[59] Hamm LL, Feng Z, Hering-Smith KS. Regulation of sodium transport by ENaC in the kidney. Curr Opin Nephrol Hypertens. 2010;19(1):98–105. 19996890 10.1097/MNH.0b013e328332bda4 PMC2895494

[60] Diakov A, Bera K, Mokrushina M, Krueger B, Korbmacher C. Cleavage in the {gamma}-subunit of the epithelial sodium channel (ENaC) plays an important role in the proteolytic activation of near-silent channels. J Physiol. 2008;586(19):4587–4608. 18669538 10.1113/jphysiol.2008.154435 PMC2614049

[61] Anand D, Hummler E, Rickman OJ. ENaC activation by proteases. Acta Physiologica. 2022;235(1):e13811. 35276025 10.1111/apha.13811 PMC9540061

[62] Wen L, Li Y, Li S, Hu X, Wei Q, Dong Z. Glucose metabolism in acute kidney injury and kidney repair. Front Med. 2021;8(11):744122. 10.3389/fmed.2021.744122 PMC8666949 34912819

[63] Araujo M, Wilcox CS. Oxidative stress in hypertension: role of the kidney. Antioxid Redox Signal. 2014;20(1):74–101. 23472618 10.1089/ars.2013.5259 PMC3880923

[64] Cowley AW Jr., Abe M, Mori T, O’Connor PM, Ohsaki Y, Zheleznova NN. Reactive oxygen species as important determinants of medullary flow, sodium excretion, and hypertension. Am J Physiol Renal Physiol. 2015;308(3):F179–F197. 25354941 10.1152/ajprenal.00455.2014 PMC4312962

