# Supplementary material for: Sex Differences in Renal Mitochondrial Respiration and H2O2 Emission in Young Dahl Salt-Sensitive Rats
Source: Function (Oxf). 2025 Oct 7;6(6):zqaf045. doi: 10.1093/function/zqaf045 (PMC12560232; doi:10.1093/function/zqaf045)
Supplement: zqaf045_Supplemental_File [file zqaf045_supplemental_file.docx]

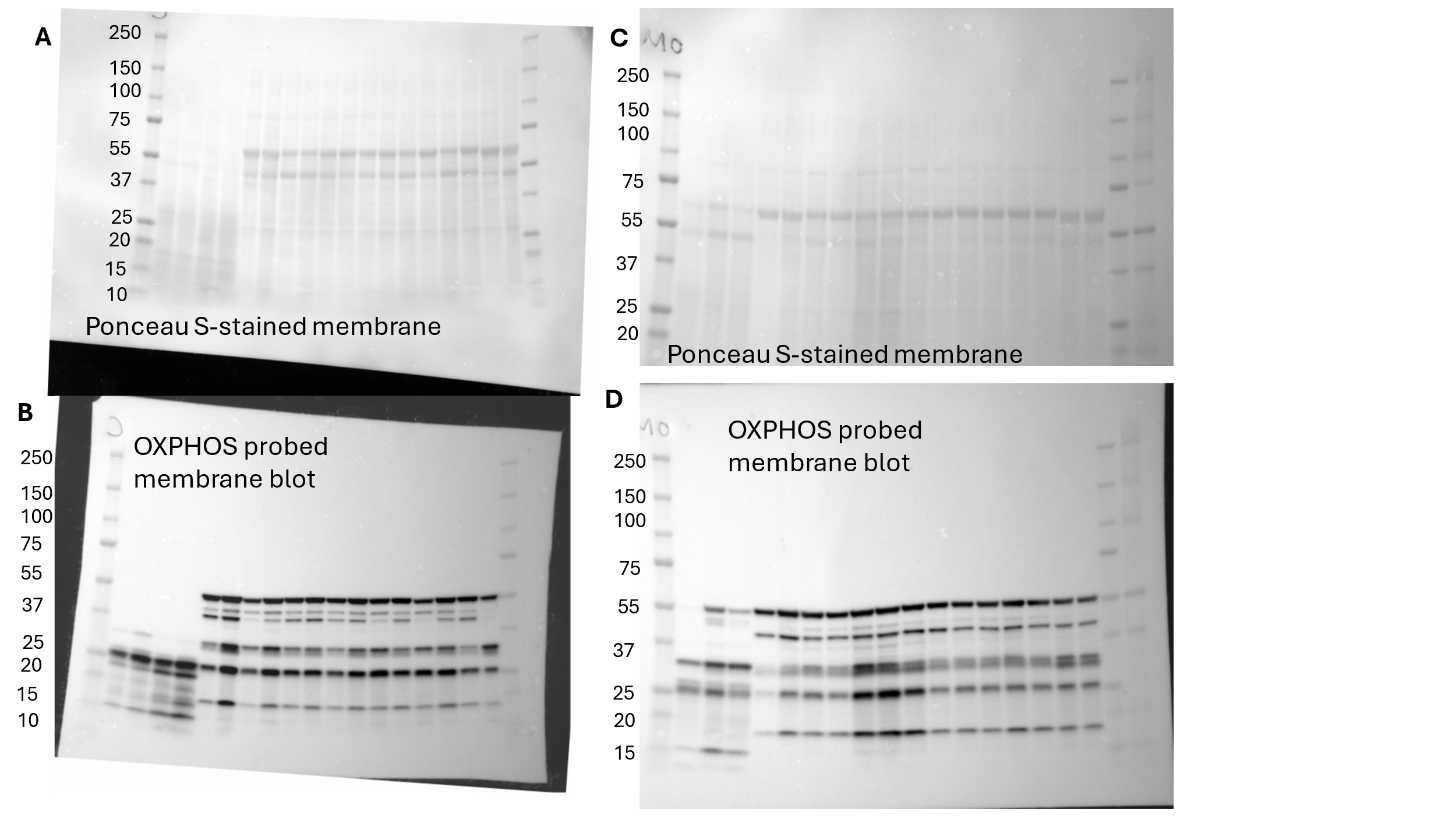


**Supplemental Figure 1: Raw Western Blots of Mitochondrial Samples from Cortex and Outer Medulla.**

This figure displays the raw, uncropped Western blots of isolated mitochondrial samples. The left panel shows samples from the renal cortex (C), while the right panel shows samples from the outer medulla (OM). Figures A and C show the Ponceau S-stained membranes, which are used to confirm equal protein loading in each lane. Figures B and D show the blots probed with an anti-OXPHOS antibody cocktail, which recognizes key proteins of the oxidative phosphorylation system.


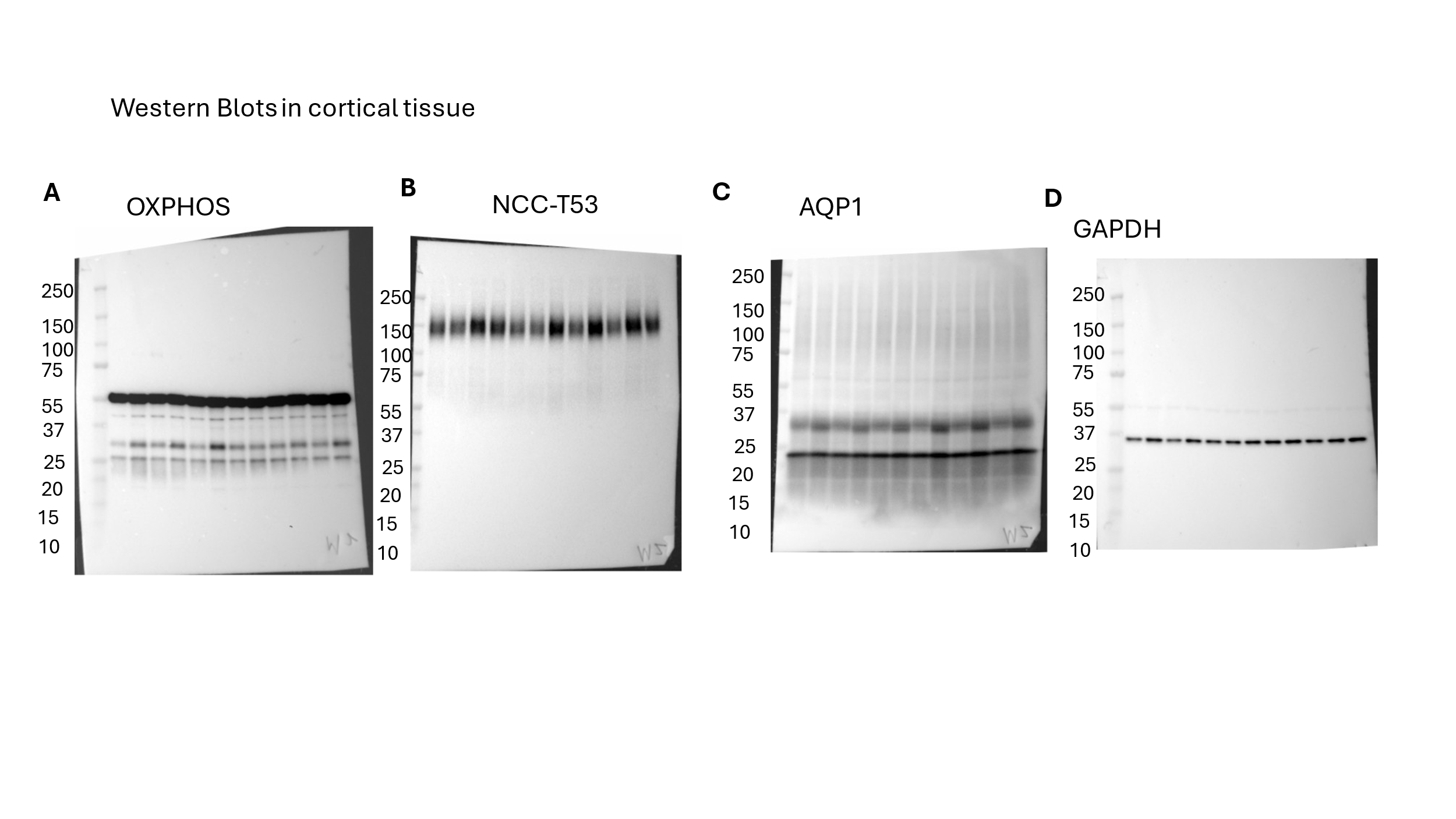


**Supplemental Figure 2: Raw Western Blots of Cortical Homogenate.**

This figure presents the raw, unedited Western blots from the renal cortical homogenate. The blots were probed with antibodies for the following proteins: A) OXPHOS; B) NCC-T53; C) AQP1; D) GAPDH.


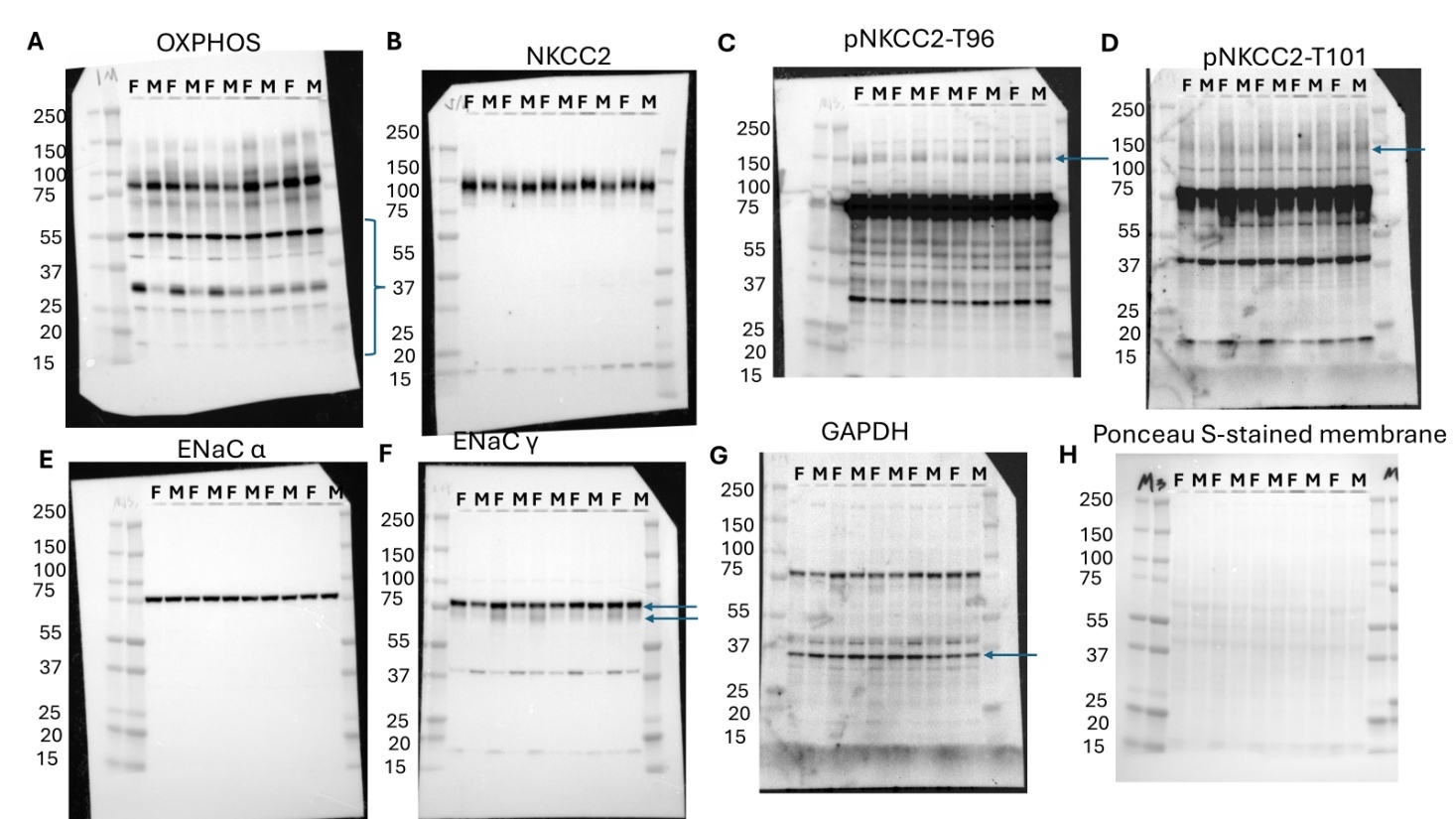


**Supplemental Figure 3: Raw Western Blots of OM Homogenate.**

This figure provides the raw, uncropped Western blots from the renal outer medulla (OM) homogenate. The blots were probed with various antibodies to assess protein abundance. A) OXPHOS; B) NKCC2; C) pNKCC2-T96; D) pNKCC2-T101; E) ENaC α; F) ENaC γ; G) GAPDH and H) Ponceau-S attained membrane. In cases where multiple bands were present, an arrow indicates the specific protein of interest.


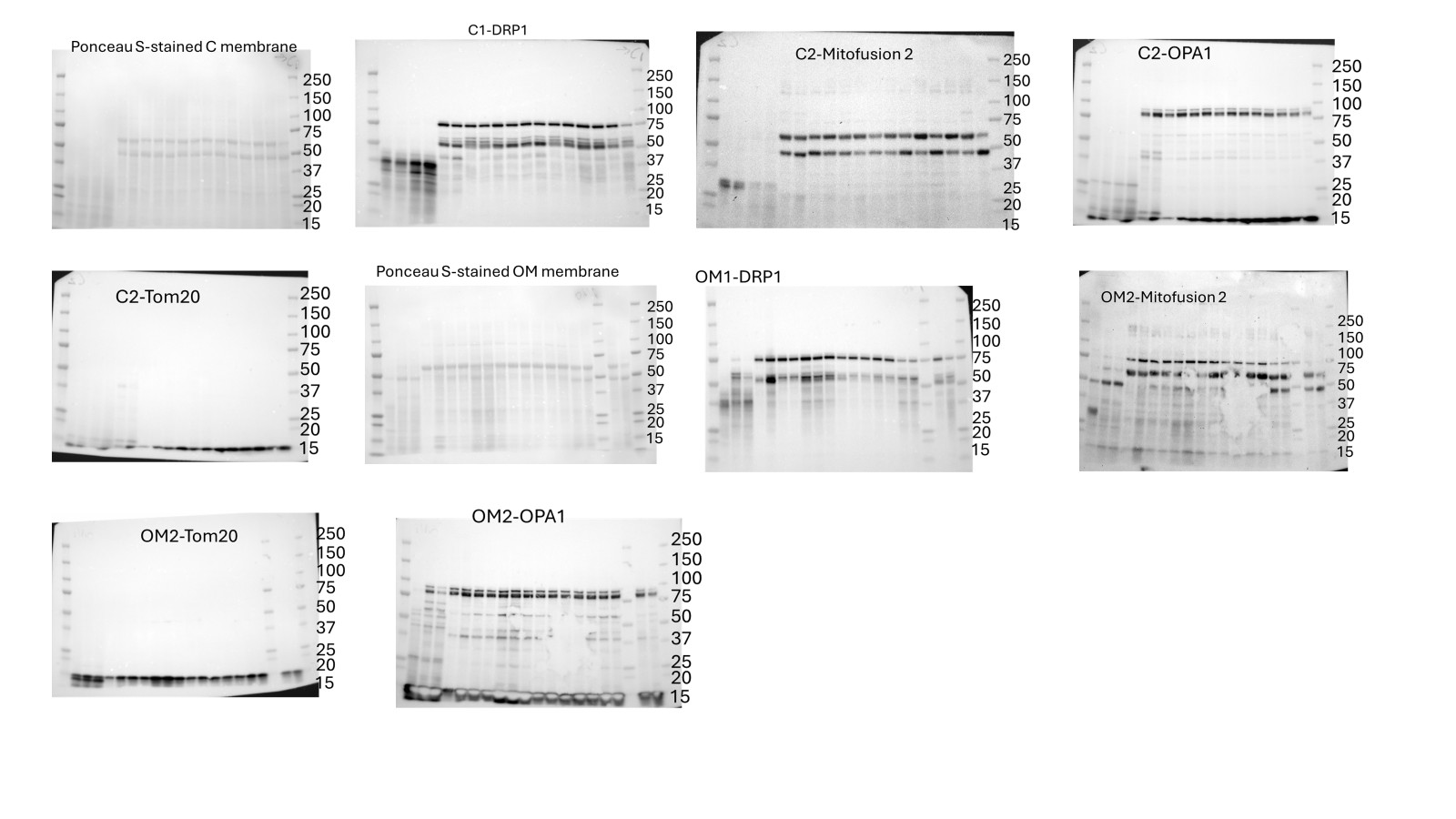
**Supplemental Figure 4: Raw Western Blots of Mitochondrial Fission and Fusion Marker Proteins.**

This figure presents the uncropped, raw Western blots for key marker proteins of mitochondrial dynamics. The blots show isolated mitochondrial samples from both the renal cortex (C) and the outer medulla (OM). The proteins probed were DRP1, OPA1, Tom20, and Mitofusion2.

**Supplemental Table 1: Two-Way ANOVA Analysis of Oxygen Consumption and H₂O₂ Emission Across Tissues.**

This table presents the results of a two-way ANOVA comparing mitochondrial function between different kidney tissues. The analysis showed that mitochondria from the outer medulla (OM) exhibited significantly higher levels of both oxygen consumption and H₂O₂ emission compared to mitochondria from the cortex.
